# A novel *Ambn-IRESCre* mouse line allows ameloblast-specific *Smad4* silencing

**DOI:** 10.3389/fphys.2026.1792453

**Published:** 2026-06-03

**Authors:** Rucha Arun Bapat, Yanbin Ji, Marziyeh Aghazadeh, Alexis E. Bauer, David C. Evans, Joseph G. Hacia, Yan Zhou, Michael L. Paine

**Affiliations:** 1 Center for Craniofacial Molecular Biology, Herman Ostrow School of Dentistry of the University of Southern California, Los Angeles, CA, United States; 2 Department of Cancer Biology, Keck School of Medicine of the University of Southern California, Los Angeles, CA, United States; 3 Department of Biomedical Sciences, Herman Ostrow School of Dentistry of the University of Southern California, Los Angeles, CA, United States

**Keywords:** ameloblast, ameloblastin, enamel, *IRES-Cre*, *Smad4* knockout mice

## Abstract

**Introduction:**

For more than two decades, enamel researchers have primarily used the keratin-14-Cre (*Krt14-Cre*) driver line to study ameloblast-specific molecular activities. Keratin-14 is expressed in multiple tissues apart from ameloblasts; thus, the use of *Krt14-Cre* to study post-birth events of amelogenesis has limitations. Therefore, to specifically study various gene functions during amelogenesis, we developed a novel *Ambn-IRESCre* mouse line that expresses Cre-recombinase only in ameloblastin-expressing cells.

**Methods and results:**

*Cre* RNA expression visualized by *in situ* hybridization closely matched *Ambn* RNA localization in *Ambn-IRESCre^+^* incisors and molars. Using two reporter lines, namely, mTmG and LacZ, we confirmed robust Cre-mediated recombination in ameloblast. We used the *Ambn-IRESCre^+^* mice as a platform to study conditional *Smad4* gene silencing mirroring the *Ambn* gene expression profile. *Smad4* is a transcription factor expressed in all cell types and inhibits epithelial cell proliferation, but its role in post-birth amelogenesis has not been studied due to neonatal lethal phenotype in *K14Cre–Smad4* conditional knockout models. We examined the *Ambn-IRESCre^+^/Smad4^fl/fl^* mice at 8 weeks and found ameloblast dysmorphology, enamel hypomineralization, lack of rod–interrod structure, and enamel loss from the surface of the incisor in otherwise healthy and viable mice.

**Discussion:**

Overall, the *Ambn-IRESCre* mouse model has been created to study amelogenesis and offers advantages over previously used Cre-recombinase models. Data collected from *Ambn-IRESCre^+/-^/Smad4^fl/fl^* mice demonstrate efficient targeting of *Smad4* in ameloblast and support the utility of this model for studying gene function during enamel formation.

## Introduction

Rodent enamel formation involves two major functional stages: secretory and maturation (Lacruz et al., 2017; Smith, 1998). Historically, amelogenesis-related gene activities have been studied using the keratin-14-driven Cre-recombinase (*Krt14-Cre*) mouse line (Eckstein et al., 2017; Furukawa et al., 2017; Huang et al., 2010; Huang et al., 2011; Soriano, 1999; Takamori et al., 2008; Xie et al., 2016). Krt14 is expressed in multiple epithelial organs including skin (Coulombe et al., 1989), oral mucosa, salivary glands, tongue (Su et al., 1993a; Su et al., 1996; Su et al., 1993b), bronchial epithelia (Rock et al., 2009), and trachea (Cole et al., 2010), which makes the *Krt14-Cre* mouse line a less-than-ideal model to study amelogenesis. So far, three amelogenin-promoter-based transgenic Cre-recombinase mouse lines have been developed, namely: *bAmelx-Cre* (Cho et al., 2013), *mAmelx-Cre* (Fan et al., 2018), and *Amelx-iCre* (Said et al., 2019). However, all three showed some variability in their transgenic Cre expression profiles within and outside the enamel organ. These non-ideal outcomes of *Krt14-Cre*, *bAmelx-Cre*, *mAmelx-Cre*, and *Amelx-iCre* to study amelogenesis thus prompted the development of a novel mouse strain with targeted insertion of Cre-recombinase driven by the endogenous ameloblastin (*Ambn*) promoter. To ensure the normal expression of *Ambn* during amelogenesis, we used the IRESCre DNA cassette (Michael et al., 1999) inserted immediately after the *Ambn* stop codon, allowing for the expression of Ambn and Cre as two independent proteins Hennecke et al., 2001.

*In situ* hybridization was used to visualize the expression of *Ambn* and *Cre* RNA. Direct Cre-mediated recombination was examined by crossing *Ambn-IRESCre* mice with mT/mG mice (Muzumdar et al., 2007). The spatiotemporal expression profile of Cre-recombinase was also observed by LacZ staining at three different timepoints in *Ambn-IRESCre^+^/R26R^+^* mice. Both *Ambn-IRESCre^+/-^* and *Ambn-IRESCre^+/+^* mouse mandibles were examined to determine any deleterious effects of the *Cre* insertion. Furthermore, based on a prior report that studied the enamel of embryonic day 11.5 (E11.5) *Krt14-Cre^+/-^/Smad4^fl/fl^* mice (Huang et al., 2010; Ko et al., 2007), we created *Ambn-IRESCre^+/-^/Smad4^fl/fl^* mice and examined their enamel. Smad4 is a transcription factor that responds to the members of the transforming growth factor β (TGF-β) superfamily that, among other functions, is involved with the regulation of cell proliferation (Heldin and Moustakas, 2012; Wrana and Attisano, 2000). The *Krt14-Cre^+/-^/Smad4^fl/fl^* mice die at birth (Huang et al., 2010; Ko et al., 2007), eliminating any possibility of *in vivo* post-natal data collection. The adult *Ambn-IRESCre^+/-^/Smad4^fl/fl^* mice were normal, except for an extreme phenotype limited to the enamel.

## Materials and methods

### Animal models

Animal use in this study was approved by the University of Southern California’s Institutional Animal Care and Use Committee (IACUC) and complies with ARRIVE 2.0 guidelines. Animals without a dental phenotype were maintained on a regular pellet diet. Animals with severe enamel phenotypes were provided with powdered food. A humane endpoint was established to avoid pulp exposure and pain in the mutant mice.

The following mouse models are referenced in this study: newly generated *Ambn-IRESCre* mice (C57BL/6J-*Ambn^em1(cre)Mlp^*/Mmjax; MMRRC strain #: 067446-JAX), mT/mG mice (B6.129(Cg)-*Gt(ROSA)26Sor^tm4(ACTB-tdTomato, -EGFP)Luo^*/J; Strain #:007676; Muzumdar et al., 2007) having cell-membrane-localized tdTomato (mT) fluorescence before recombination and EGFP (mG) fluorescence expression after Cre-recombination; R26R mice (B6.129S4-*Gt(ROSA)26Sor^tm1Sor^*/J; JAX strain #: 003474; Soriano, 1999) harboring a *loxP*-flanked DNA STOP sequence preventing the expression of a downstream *LacZ* reporter gene; and *Smad4* floxed mice (STOCK *Smad4^tm2.1Cxd^*/J; JAX strain #: 017462; Yang et al., 2002) to conditionally delete *Smad4* after crossing with *Ambn-IRESCre* mice.

### Development of the *Ambn-IRESCre* mouse line

CRISPR/Cas9 technology was used to generate the *Ambn-IRESCre* driver mouse line at the Jackson Laboratories (USA). *Cre*-recombinase was inserted after the *Ambn* stop codon following the internal ribosomal entry site (IRES) (Michael et al., 1999) to assure no disruption to the expression profile of Ambn. Genomic DNA was isolated from both the founder mice (F1) and the first backcross generation (N1), and the entire inserted region was PCR-amplified by long range PCR (**Supplementary Figure 1**) and sequenced to confirm that there were no DNA sequence errors with the knock-in strategy. Genotype confirmation was obtained by probe PCR. The long-range PCR primers are listed in **Supplementary Figure 1**, and the probe PCR primers are listed in **Supplementary Table 1**.

### Genotyping protocols

The PCR primer sets for each genotyping PCR are listed in **Supplementary Table 1**. MMRRC or JAX protocols for standard PCR were followed for genotyping.

### *In situ* hybridization to detect *Ambn* and *Cre* RNA

Post-natal (PN) 14-day-old mandibles were harvested from WT, *Ambn-IRESCre^+/-^*, and *Ambn-IRESCre^+/+^* mice. After ~24 h of fixation in 10% neutral buffered formalin at room temperature, the samples were decalcified in 10% EDTA for ~3 weeks at 4 °C, and 6-μm-thin sections were prepared from paraffin blocks made using standard dehydration and embedding protocols for formalin-fixed, paraffin-embedded sections (FFPE). ISH was performed using the commercially available *Ambn* (catalog #508241) and *Cre* (catalog #312281) probes and RNAscope 2.5 Assay RED HD Detection Reagent kit (ACD Bio) by following the FFPE Sample Preparation and Pretreatment protocol (document numbers 322452, 322360-USM). Incubation times, probe volumes, and imaging conditions were consistent among WT, *Ambn-IRESCre^+/-^*, and *Ambn-IRESCre^+/+^* samples and probes. Serial sections from the same paraffin block were used for *Ambn* and *Cre* ISH experiments to accurately depict their localization.

### *Cre-*mediated recombination detection in *Ambn-IRESCre/mTmG* mice

PN14 mandibles were harvested from WT and *Ambn-IRESCre^+/-^/mTmG^+/-^* animals, and 6-μm-thin FFPE sections were made as detailed above. A previously published immunofluorescence protocol by Liang et al. (2022) was followed to label the sections with anti-GFP antibody conjugated with Alexa Fluor^®^ 488 at 1:500 dilution (Jackson ImmunoResearch #300-545-245) to detect GFP signal after Cre-recombination. Imaging was performed using a Leica Stellaris confocal microscope.

### *In vivo* β-galactosidase detection by LacZ staining

PN3-, PN5-, and PN8-days-old mandibles from mice with the genotype of *Ambn-IRESCre^+/-^/R26R^+/-^* were harvested as samples, and *R26R^-/-^* mandibles were used as controls. The mandibles were dissected and fixed in 0.2% glutaraldehyde at 4 °C overnight and prepared for β-galactosidase (lacZ) staining using protocols described by Hu et al. (2008) and by Liang et al. (2019). Throughout the staining procedure, 2 mM MgCl_2_ was added to all solutions to maintain the activity of the β-galactosidase enzyme. LacZ activity was detected by using X-Gal staining kit from OZ Biosciences (catalog #GX10003). Brightfield microscopic images were captured using Keyence BZ-X710 microscope.

### Micro-computed tomography

Eight-week-old WT, *Ambn-IRESCre^+/-^*, *Ambn-IRESCre^+/+^*, and *Ambn-IRESCre^+^/Smad4^fl/fl^* mandibles were used for the µCT analysis. Micro-CT protocol from Eckstein et al. (2017) was followed with slight modifications. The mandibles were dissected, fixed with 4% paraformaldehyde, and scanned using SkyScan 1174 µCT (Bruker) at 14.12-µm resolution, 50 kV voltage, and 800 µA current. The system was calibrated using 0.25 and 0.75 g/cm^3^ hydroxyapatite phantoms. Relative mineral densities of the mandibular incisor enamel and dentin were analyzed using CTan software (version 1.14.4). Minimal animal numbers that allow statistical analysis were included. Incisor µCT sections in the coronal plane at the following anatomical landmarks were used to calculate the enamel and dentin mineral densities: (1) at the edge of the labial cortical bone just after eruption, (2) mesial to the mesial cusp of the first molar, and (3) distal to the distal cusp of the first molar to roughly represent the late maturation and mid- and early maturation stages of enamel formation, respectively (Bui et al., 2023). Five slices were averaged at each anatomical landmark for determining mineral density. For enamel mineral density, regions of interest (ROIs) were drawn freehand to completely cover the enamel at each anatomical landmark. For incisor dentin mineral density, two 10 × 10 pixel square ROIs were placed within the bulk of the incisor dentin equidistant from the pulp and DEJ.

For the *Ambn-IRESCre^+/-^/Smad4^fl/fl^* samples, three-dimensional reconstruction, dentin and enamel segmentation, and dentin and enamel volume quantifications were performed using Amira/Avizo software (ThermoFisher Scientific, USA). Enamel and dentin were segmented separately using tissue-specific masking-threshold values. In WT samples, the automated enamel segmentation was accurate and was verified slice by slice for each sample. In contrast, the automated segmentation of mutant enamel, as well as WT and mutant dentin, frequently included adjacent bone tissue. Therefore, these segmentations were manually corrected slice by slice for each sample. For the first molar dentin mineral density, two landmarks were used—within the mesial cusp, in a line drawn tangential to the mesial root of the first molar, and in the central cusp, in a line drawn through the furcation. A 10 × 10-pixel square region of interest was chosen equidistant from both the pulp and DEJ.

### Scanning electron microscopy and back-scatter SEM

Scanning electron microscopy (SEM) and back-scatter SEM (BS-SEM) were performed to determine the microstructure of 8-week-old incisor enamel using FEI Nova Nano SEM (voltage 10 kV and spot size 4) at the USC Core Center of Excellence in Nano Imaging according to previously published protocols (Huang et al., 2011). One hemimandible from each mouse previously used for µCT was stored dry at 4 °C and used for SEM analysis. The incisors were fractured in the coronal plane just prior to eruption using a diamond saw to expose the rod–interrod architecture. Fractured surfaces were polished and etched with 30% phosphoric acid for 40–45 s before sputter coating with platinum/palladium to make them conductive. *Ambn-IRESCre^+/-^/Smad4^fl/fl^* mutant incisors were only etched for ~10 s to prevent the loss of mutant enamel.

### Histology and immunofluorescence

The FFPE sections of PN14 *Ambn-IRESCre^+/+^* mandibles were prepared as described earlier (*in situ* hybridization section) for hematoxylin and eosin (H&E) staining to observe the ameloblast morphology (Lacruz et al., 2012). Amelogenin and ameloblastin proteins were co-immunolabeled within the ameloblasts and enamel matrix using primary antibodies against mouse amelogenin (a gift from Dr. M. Snead, 1:1,000 dilution) and ameloblastin (M300 antibody, Santa Cruz Biotech or R&D systems #AF3026, 1:500 dilution) using previously established protocol (Bapat et al., 2020).

Eight-week-old *Ambn-IRESCre^+/-^/Smad4^fl/fl^* and WT control mandibles previously used for µCT were decalcified in 10% EDTA at 4 °C for 3 to 4 weeks. Sample processing, sectioning, H&E, and immunofluorescence (IF) protocols for amelogenin and ameloblastin co-labeling were the same as above. Cytokeratin-14 (Proteintech, #10143-1-AP, 1:200) and Smad4 (Invitrogen #PA5-34806, 1:100) immunolabeling was performed on FFPE sections using previously published protocol (Liang et al., 2022). Imaging was performed using a Leica Stellaris confocal microscope.

### Transcript analysis by polymerase chain reaction

*Ambn* has 2 isoforms differing in size by 45 base pairs. Isoform 1 is longer, containing 1,269 nucleotides (NM_001303431.1), and isoform 2 is shorter (NM_009664.2), having 1,224 nucleotides. To validate that the insertion of *Cre* in the 3′ untranslated region (3′ UTR) of *Ambn* did not affect its alternative splicing, we quantified the ratio and volumes of the *Ambn* isoforms using PCR and nucleotide gel electrophoresis in WT, *Ambn-IRESCre^+/-^*, and *Ambn-IRESCre^+/+^* animals. RNA was isolated from PN14 first molar enamel organs using Trizol, and cDNA was synthesized using Maxima™ H Minus cDNA Synthesis Master Mix, with dsDNase (ThermoFisher Scientific, USA) using the manufacturer’s protocol. Primers were designed (**Supplementary Table 2**, primer *Ambn*^#^) to amplify a segment covering the alternatively spliced region, and conventional PCR was performed using GoTaq^®^ PCR Master Mix (Promega, USA). PCR products were visualized using 2% agarose gel containing SYBR™ Safe DNA gel stain (ThermoFisher Scientific, USA). Similar experiments were conducted to visualize *Amelx* and *Odam* transcripts using WT, *Ambn-IRESCre^+/-^*, and *Ambn-IRESCre^+/+^* cDNA as template and primers as listed in **Supplementary Table 2**. For quantification, band volumes were measured using Azurespot image analysis software (Azure Biosystems, Dublin, CA, USA) and normalized against *β-actin* as loading control (primers shown in **Supplementary Table 2**). Statistical analysis was performed using unpaired *t*-tests assuming unequal variances with three different samples in each genotype.

### Quantitative PCR for selected gene profiling

Mandibular first molar enamel organs were harvested for gene expression analysis from PN14 *Ambn-IRESCre^+/-^/Smad4^fl/fl^* and age-matched littermate controls. The qPCR conditions were 95 °C for 2 min, followed by 95 °C for 15 s and 62 °C for 1 min repeated for 40 cycles (Sarkar et al., 2014). The qPCR primers are listed in **Supplementary Table 2**.

RNA isolation and cDNA synthesis were conducted as detailed above. PowerUp™ SYBR™ Green Master Mix (ThermoFisher Scientific, USA) was used for qPCR. Fold change in gene expression was calculated for *Amelx*, *Ambn*, *Odam*, *Smad4*, and *Cre* with *β-actin* as internal control in three independent experiments (biological replicates) using the 2^-ΔΔCt^ method. Each reaction was performed in quadruplets, and calculation of fold change values and statistical analysis of the gene expression data were conducted using technical replicates.

### Statistical analysis

All statistical analyses were conducted using Microsoft Excel version 2209. Mean ± standard deviation (SD) is shown in enamel volume, dentin surface area, and dentin volume graphs. Percent enamel mineral density and dentin mineral density were calculated by assigning 100% to the most highly mineralized enamel or dentin measurement, respectively, and normalizing the rest to it. The mineral density graphs depict mean percent density ± normalized standard deviation. For transcript analysis, band volumes were normalized against *β-actin* internal control, and the mean ± standard deviation (SD) values of three samples in each genotype are represented in the graphs. The graphs were designed in GraphPad Prism version 9.4.1 or in Microsoft Excel version 2209. All statistical analyses were conducted in Microsoft Excel. *P*-values were calculated by using pairwise unpaired two-sample *t*-tests assuming unequal variances, unless the variances were identical for both test and control groups, in which case two-sample *t*-tests assuming equal variances were performed. *P* ≤0.05 was considered statistically significant (*p ≤ 0.05, **p ≤ 0.01, ***p ≤ 0.001).

## Results

### Localization of *Ambn* and *Cre* RNA in *Ambn-IRESCre* mice is as intended

**Figure 1:**
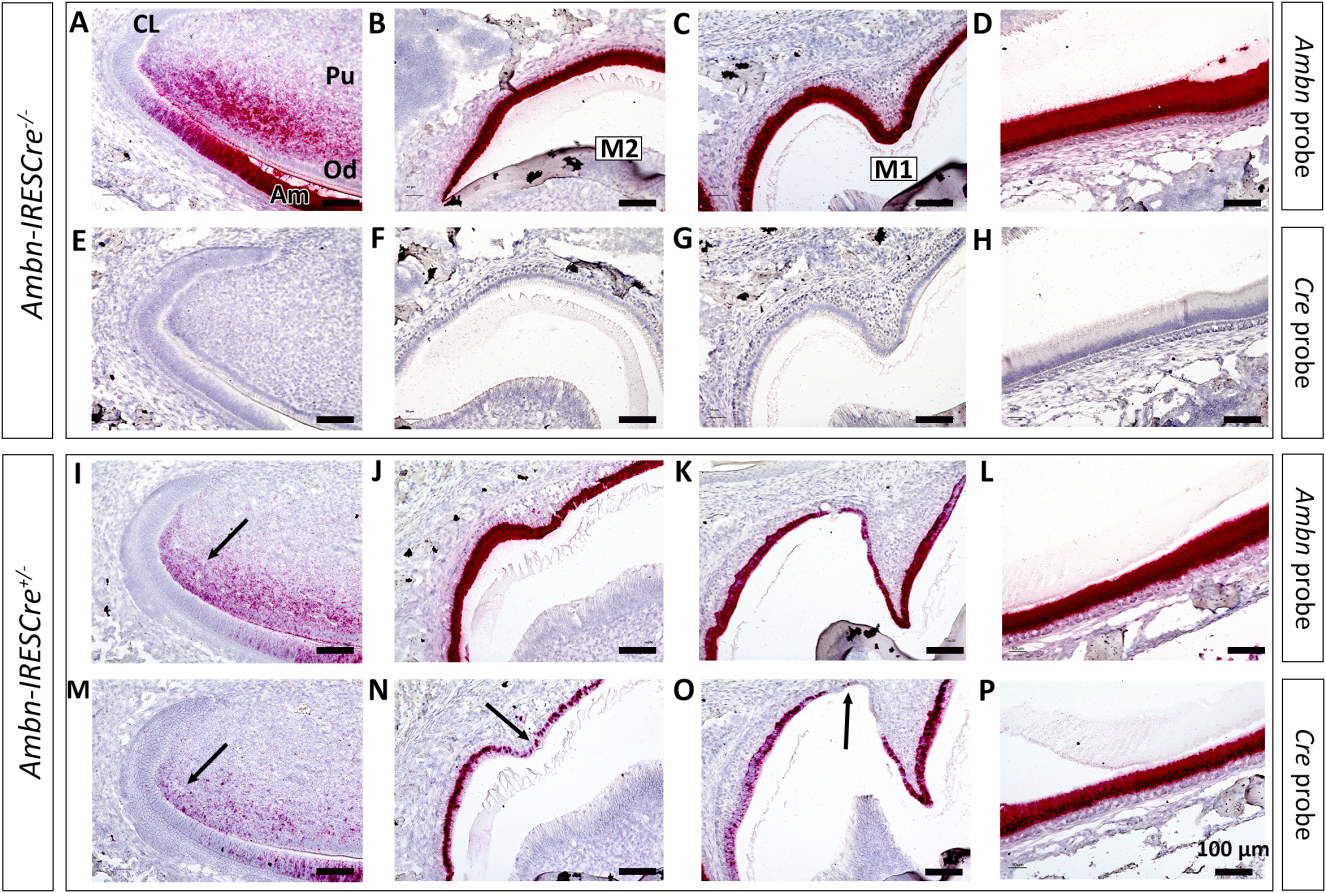
Brightfield microscopic images showing *in situ* hybridization of *Ambn* and *Cre* RNA in PN14 *Ambn-IRESCre^-/-^* (WT) and *Ambn-IRESCre^+/-^* incisors and molars. The earliest appearance of *Ambn* RNA in WT incisors was seen in the pulp just anterior to the cervical loop in pre-odontoblasts and in pre-ameloblasts **(A)**. Robust *Ambn* expression was seen in the second molar [M2, **(B)**], first molar [M1, **(C)**], and secretory-stage incisor ameloblasts **(D)**. There was no *Cre* signal observed in the WT incisor or molars **(E–H)**. *Ambn* expression in the *Ambn-IRESCre^+/-^* incisor was identical to that of WT in the earliest pulp and pre-odontoblasts near the cervical loop as well as pre-ameloblasts [**(I)**, arrow]. *Ambn-IRESCre^+/-^* second **(J)** and first **(K)** molars as well as incisor secretory stage ameloblast **(L)** showed the intensity and localization of *Ambn* RNA similar to WT. The *Cre* RNA expression pattern in *Ambn-IRESCre^+/-^* incisors closely followed the distribution of *Ambn* RNA. In the pulp and pre-odontoblast [**(M)**, arrow] as well as late-maturation-stage ameloblast in the molars [**(N, O)** arrows], its intensity was less compared to *Ambn* RNA. In secretory-stage incisor ameloblast, both the intensity and localization of *Cre* RNA were identical to that of *Ambn* RNA **(P)**. CL, cervical loop; Pu, pulp; Od, odontoblasts; Am, ameloblasts.

To directly examine the expression pattern of *Ambn* and *Cre* RNA, we used *in situ* hybridization at PN14. In WT mandibles, strong *Ambn* expression was observed in incisor and molar ameloblasts as expected (**Figures 1A–D**; **Supplementary Figure 2A**). *Ambn* RNA was also expressed in the incisor pulp anterior to the cervical loop, in the pre-odontoblasts (**Figure 1A**), as well as at the root apices of the first and second molars (**Supplementary Figure 2A**). There was no *Cre* signal in the WT samples (**Figures 1E–H**; **Supplementary Figure 2B**).

The localization and intensity of *Ambn* RNA in *Ambn-IRESCre^+/-^* incisors and molars were unchanged compared to WT (**Figures 1I–L**; **Supplementary Figures 2C, E**). Like WT, *Ambn* signal was present in *Ambn-IRESCre^+/-^* pulp tissue just anterior to the cervical loop (**Figure 1I**, arrow, **Supplementary Figure 2C**). *Ambn-IRESCre^+/-^* ameloblasts showed a consistent *Ambn* expression in both incisor and molars until the maturation stage (**Figures 1J–L**). *Ambn* expression in odontoblast continued weakening anteriorly (**Supplementary Figures 3A, B**) such that mature odontoblasts and pulp had no significant *Ambn* RNA (**Supplementary Figures 3C, D**).

The intensity and localization of *Cre* RNA in *Ambn-IRESCre^+/-^* ameloblasts matched the *Ambn* expression pattern very closely, starting weakly in pre-ameloblast and strengthening in secretory ameloblast (**Figures 1P**; **Supplementary Figures 2D, F**). *Cre* RNA expression was significantly weaker compared to *Ambn* RNA in the pre-odontoblast, pulp, and molar root apices (**Figure 1M**, arrow, **Supplementary Figures 3E, F**, arrows). Specifically, every pre-odontoblast that was positive for *Ambn* RNA did not express *Cre* RNA as detected by serial sections (**Figures 1I, M**, arrows). *Cre* expression was absent in mature odontoblasts and pulp tissue (**Supplementary Figures 3G, H**). The intensity of *Cre* RNA was also weaker than *Ambn* in late-maturation-stage ameloblasts in molars as well as incisors (**Figures 1N, O**, arrows, **Supplementary Figure 4**).

*Ambn-IRESCre^+/+^* ISH revealed cysts within ameloblasts, which are described in the following sections. The cysts were lined by cells positive for both *Ambn* and *Cre* RNA (**Supplementary Figures 2G, H**, **Supplementary Figure 5**).

### GFP immunofluorescence in *Ambn-IRESCre/mTmG* shows Cre-mediated recombination predominantly in ameloblast and also in odontoblast and alveolar bone

**Figure 2:**
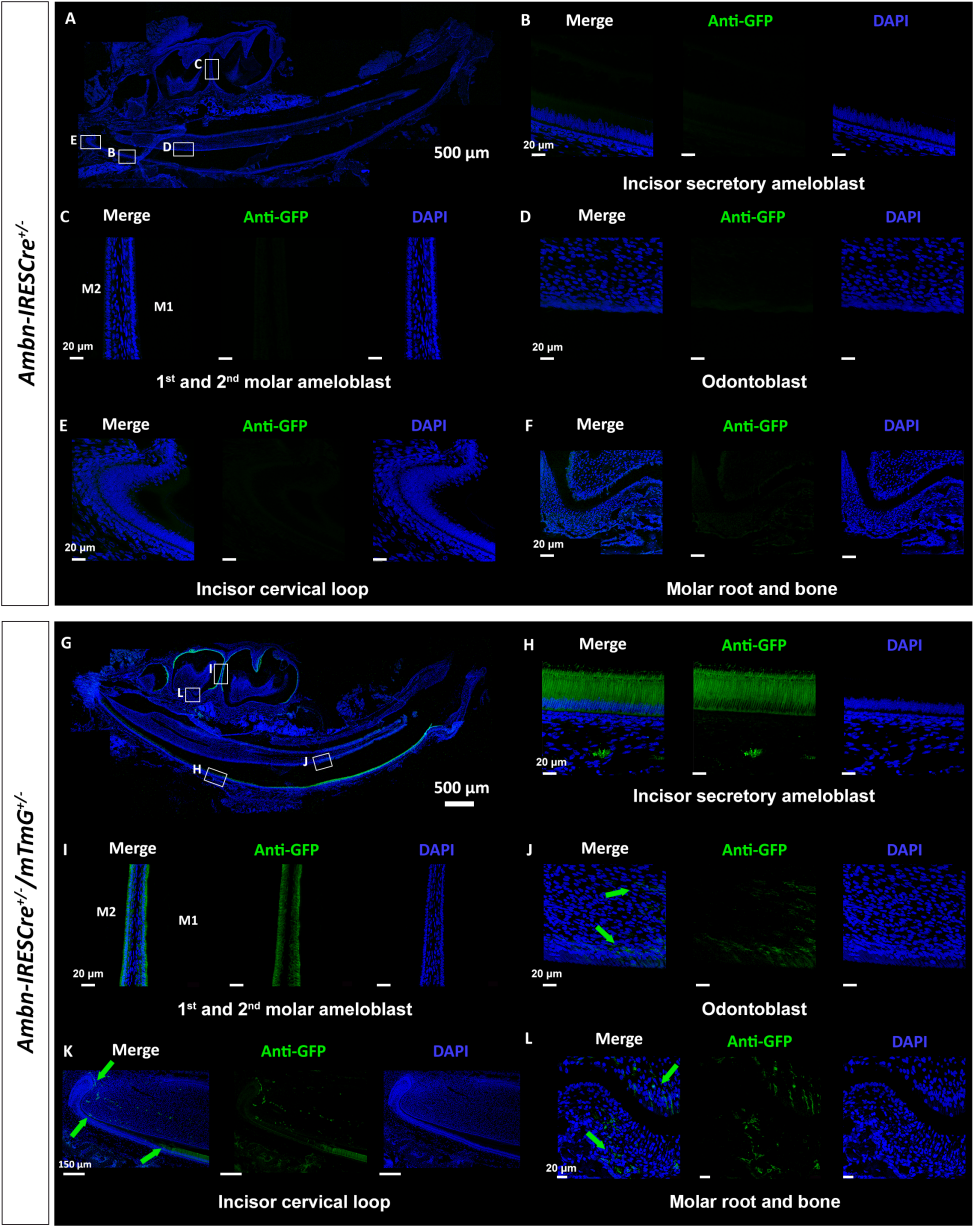
Confocal microscopic images of PN14 *Ambn-IRESCre^+/-^* control **(A–F)** and *Ambn-IRESCre^+/-^/mTmG^+/-^* **(G–L)** mandibles showing green fluorescence in cells that have undergone Cre-mediated recombination. No green fluorescence was observed in the control regions studied [**(A)**, ×10 magnification tile-scan image, **(B–F)**, ×63 magnification images; panel **(F)** is not from **(A)**]. In the *Ambn-IRESCre^+/-^/mTmG^+/-^* mandible, bright green fluorescence was observed in incisor and molar ameloblasts in the ×10 tile-scan **(G)**. In the incisor secretory stage and first and second molar maturation stage ameloblast, green fluorescence was robust **(H, I)**. Few odontoblast and pulp cells were positive for Cre-recombination [**(J)**, green arrows]. In the tile-scan of the cervical loop, few pulp cells anterior to the cervical loop and pre-odontoblast were green, while in ameloblast green fluorescence appeared in the secretory stage[**(K)**, green arrows, panel **(K)** is not from **(G)**]. Few odontoblasts within the second molar root and alveolar bone cells within the furcation region were positive for GFP [**(L)**, arrows]. M1, first molar; M2, second molar.

Validation of Cre-mediated recombination was performed in *Ambn-IRESCre/mTmG* mice by detecting GFP immunofluorescence. In *Ambn-IRESCre^+/-^* control mice, no GFP signal was observed in the incisor-secretory-stage ameloblasts, first and second molar maturation-stage ameloblasts, odontoblast, cervical loop, and molar roots and furcation area (**Figures 2A–F**). In *Ambn-IRESCre^+/-^/mTmG^+/-^* mandibles, a strong green fluorescence was observed in incisor secretory-stage ameloblasts as well as first and second molar ameloblasts in the maturation stage (**Figures 2G–I**). GFP signal was also observed in a few odontoblast and pulp cells (**Figure 2J**). The cervical loop region was specifically examined to note GFP fluorescence in pre-odontoblast and the initiation of GFP signal in ameloblast (**Figure 2K**). Pulp cells anterior to the cervical loop and pre-odontoblast just apical to pre-ameloblast were positive for GFP, while a strong green signal was initiated in ameloblasts after their transition from pre-ameloblast, which is later than the appearance of *Cre* RNA in ISH (**Figure 2K**, arrows). In the molar root and furcation area, an intermittent GFP signal was observed in root odontoblasts as well as the alveolar bone (**Figure 2L**, arrows).

### LacZ reporter activity reveals historical *Ambn-IRESCre*-mediated recombination patterns

**Figure 3:**
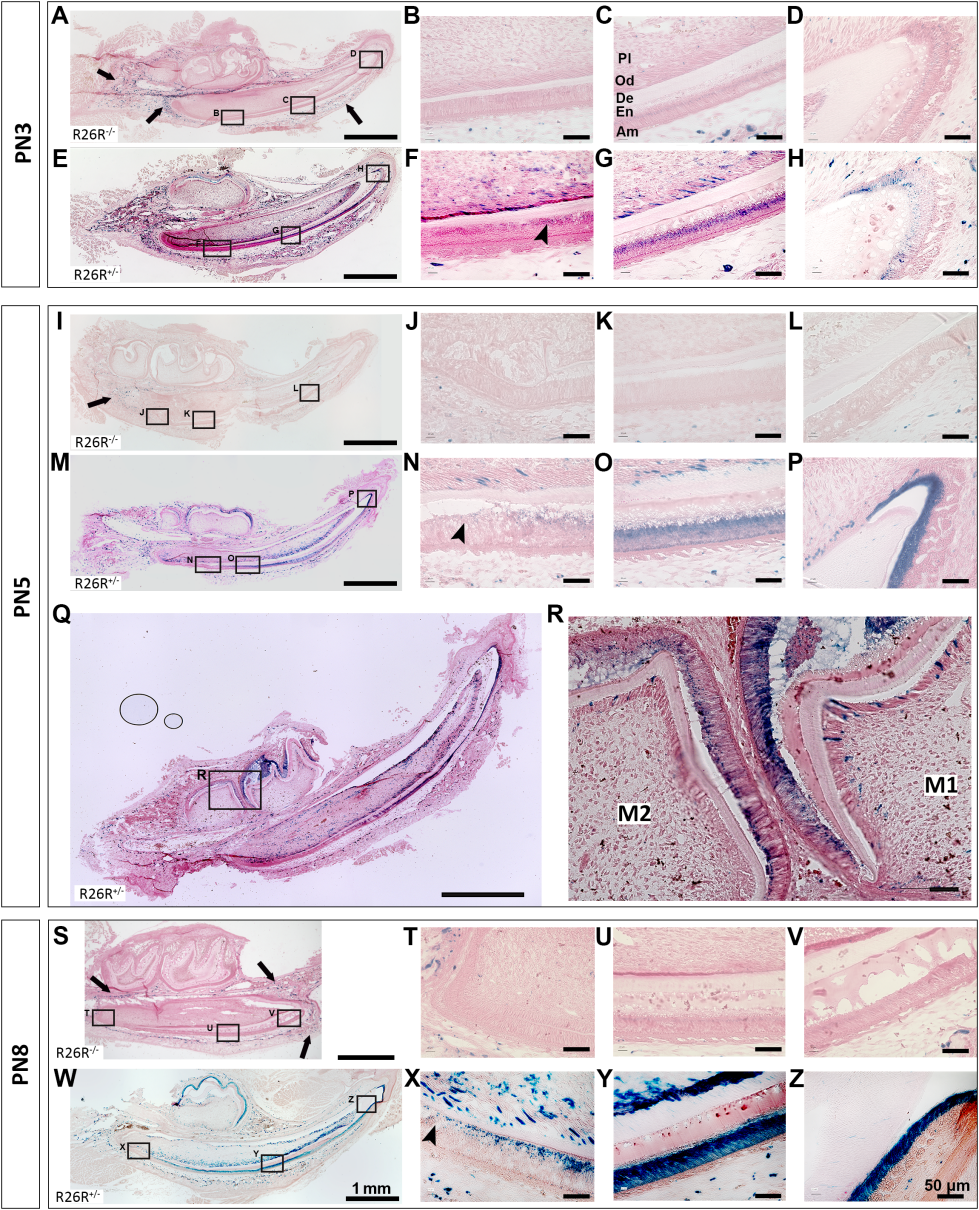
Brightfield microscopic images of 3-, 5-, and 8-day-old (PN3, PN5, and PN8) mandibles from age-matched *R26R^-/-^* (control) and *Ambn-IRESCre^+/-^/R26R^+/-^* (represented as R26R^+/-^) mice showing the expression pattern of *Cre*-recombinase as observed by LacZ staining. The post-natal-day-3 control **(A–D)** showed background staining in the bone (arrows). PN3 *R26R^+/-^* showed a similar staining in the bone along with a specific positive signal within ameloblasts **(E)**. Initiation of LacZ signal in ameloblasts was observed in PN3 *R26R^+/-^*, arrowhead in **(F)**. LacZ staining was also noted in a few pulp cells adjacent to the odontoblasts and some early odontoblasts **(E–G)**. Blue LacZ staining continued in secretory stage ameloblasts until the incisor tip **(G, H)**. At PN5, the controls only showed background stain, primarily in the bone [**(I–L)**, arrow in **(I)**]. *R26R^+/-^* ameloblasts displayed intensified LacZ staining, and some odontoblasts also appeared blue **(M–P)**. Initiation of LacZ staining is marked by an arrowhead **(N)**. At PN5, first and second molars were also positive for LacZ, which is depicted in **(Q, R)**. Weaker LacZ staining was observed in the second molar, M2 in **(R)**, compared to the more mature first molar, M1 in **(R)**. At PN8, control ameloblasts continued to remain negative for LacZ **(S–V)** but continued to show blue staining in the alveolar bone [**(S)**, arrows], while *R26R^+/-^* ameloblasts showed a strong blue staining **(W–Z)**. Initiation of LacZ staining is marked using an arrowhead in **(X)**. Some early odontoblasts, pulp cells adjacent to the odontoblasts, **(X)** and almost all mature odontoblasts appeared blue **(W–Y)**. Maturation stage incisor ameloblasts were strongly positive **(Z)**. Pl, pulp; Od, odontoblasts; De, dentin; En, enamel; Am, ameloblasts; the scale bar for the ×4 images is in **(W)**; the scale bar for the higher-magnification images is in **(Z)**.

To broaden the Cre validation, a traditional LacZ reporter system was also utilized by examining age-matched *R26R^-/-^* (control) and *Ambn-IRESCre^+/-^/R26R^+/-^* mice at PN3-, PN5-, and PN8 days old. In the control mice, blue background staining was noted in non-tooth tissues like the alveolar bone at all ages (**Figures 3A, I, S**, arrows). Initial weak LacZ staining could be noted in *Ambn-IRESCre^+/-^/R26R^+/-^* early secretory ameloblasts at PN3 (**Figures 3E, F**), PN5 (**Figures 3M, N**), and PN8 (**Figures 3W, X**). LacZ staining was also observed in the first mandibular molar as early as PN3 (**Figure 3E**) and in the second mandibular molar at PN5 (**Figure 3M**). Greater detail of LacZ expression in the mandibular first and second molars can be viewed in in **Figures 3Q, R**. The first molar (M1 in **Figure 3R**), which is at a more advanced stage of ameloblast development, showed a stronger LacZ staining when compared to the less mature second molar (M2 in **Figure 3R**). LacZ expression was also observed in some pre-odontoblasts and odontoblasts (**Figures 3F, G, O, R, X, Y**). LacZ expression continued in the transition stage (PN3, **Figure 3H**) and became stronger in maturation-stage incisor ameloblasts (**Figures 3P, Z**; PN5 and PN8).

Additional LacZ expression data for *Ambn-IRESCre^+/-^/R26R^+/-^* PN8 mice is presented in **Supplementary Figure 6**. At PN8, the first and second maxillary molars and the mandibular second molar were LacZ-positive along with the mandibular incisor; however, the mandibular third molar is LacZ-negative (**Supplementary Figure 6**, arrow). At PN8 and in all sections examined, the tongue was LacZ-negative (**Supplementary Figure 6**, arrowhead).

### *Ambn-IRESCre* phenotype

**Figure 4:**
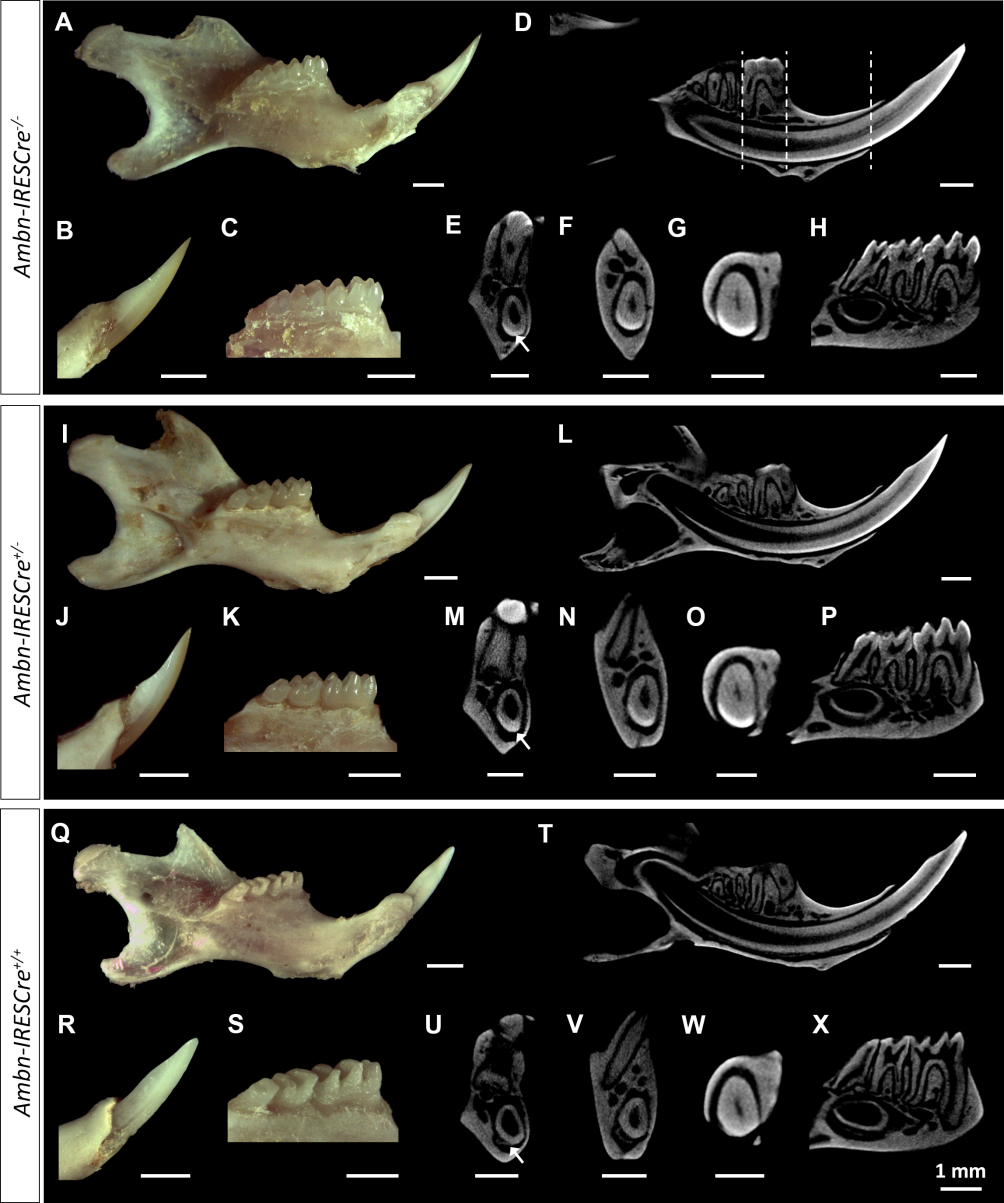
Photographic images and μCT sections of 8-week-old *Ambn-IRESCre^-/-^* (WT), *Ambn-IRESCre^+/-^*, and *Ambn-IRESCre^+/+^* mandibles. WT enamel appeared smooth and translucent on the incisor and molars **(A–C)**. μCT showed a uniform layer of enamel covering the entire length of the incisor **(D)** and molars **(H)** in the sagittal section. The dotted lines in **(D)** represent the three anatomical landmarks where coronal μCT sections of the incisor were taken for all samples: WT coronal sections at the distal to the first molar region in **(E)**, mesial to the first molar region in **(F)**, and at the labial cortical bone edge in **(G)**. The arrow in **(E)** represents the earliest appearance of mineralized enamel. *Ambn-IRESCre^+/-^* enamel appeared normal similar to WT in both photographic **(I–K)** and μCT evidence **(L–P)**. The arrow in **(M)** represents the earliest appearance of mineralized enamel distal to the first molar region, similar to WT. The *Ambn-IRESCre^+/+^* incisor was blunt **(Q, R)**, and the enamel appeared opaque white and pitted **(R, S)**. The *Ambn-IRESCre^+/+^* incisor surface appeared rough with a thin mineral layer on the surface of dentin in the sagittal μCT section **(T)**. No enamel was detected in the coronal sections [arrow in **(U)**], while some ectopic minerals were visible **(U–W)**. There was also no distinct enamel layer on *Ambn-IRESCre^+/+^* molars **(X)**.

*Ambn-IRESCre^+/-^* and *Ambn-IRESCre^+/+^* animals appeared healthy and had normal breeding patterns. The gross phenotype of 8-week-old WT (*Ambn-IRESCre^-/-^*), *Ambn-IRESCre^+/-^*, and *Ambn-IRESCre^+/+^* incisor and molar enamel was analyzed by visual inspection, µCT, and SEM. The WT (*Ambn-IRESCre^-/-^*, **Figures 4A–C**) and *Ambn-IRESCre^+/-^* (**Figures 4I–K**) incisor and molar enamel, respectively, was smooth and translucent and had no visible differences. In the sagittal and coronal µCT sections of WT (**Figures 4D–G**) and *Ambn-IRESCre^+/-^* (**Figures 4L–O**) incisors, the enamel was clearly distinguishable from the underlying dentin as a highly mineralized zone in all three anatomical landmarks studied. In the early maturation stage, distal to the first molar, the incisor enamel was identifiable as a bright white segment (**Figures 4 E, M**, arrows) which continued until eruption (**Figures 4F, G, N, O**). Enamel was visible on the lateral surfaces of WT and *Ambn-IRESCre^+/-^* molar cusps in sagittal sections (**Figures 4H, P**). Upon visual inspection, the *Ambn-IRESCre^+/+^* teeth appeared chalky white and rough (**Figures 4Q–S**). The µCT analysis revealed a thin, rough layer of mineral on the surface of *Ambn-IRESCre^+/+^* incisor (**Figure 4T**). In coronal sections, no incisor enamel was clearly visible on underlying dentin (**Figures 4U–W**), and the molar crowns had sparse areas of enamel on their surface (**Figure 4X**).

The WT and *Ambn-IRESCre^+/-^* incisor enamel mineral density as quantified by µCT was similar throughout the maturation stage in all three selected regions (**Supplementary Figure 7 A**). However, there were significant differences between the WT and *Ambn-IRESCre^+/+^* (**Supplementary Figure 7B**) as well as *Ambn-IRESCre^+/-^* and *Ambn-IRESCre^+/+^* (**Supplementary Figure 7C**) enamel mineral densities. The *Ambn-IRESCre^+/+^* incisor enamel was severely hypomineralized in all three regions examined.

**Figure 5:**
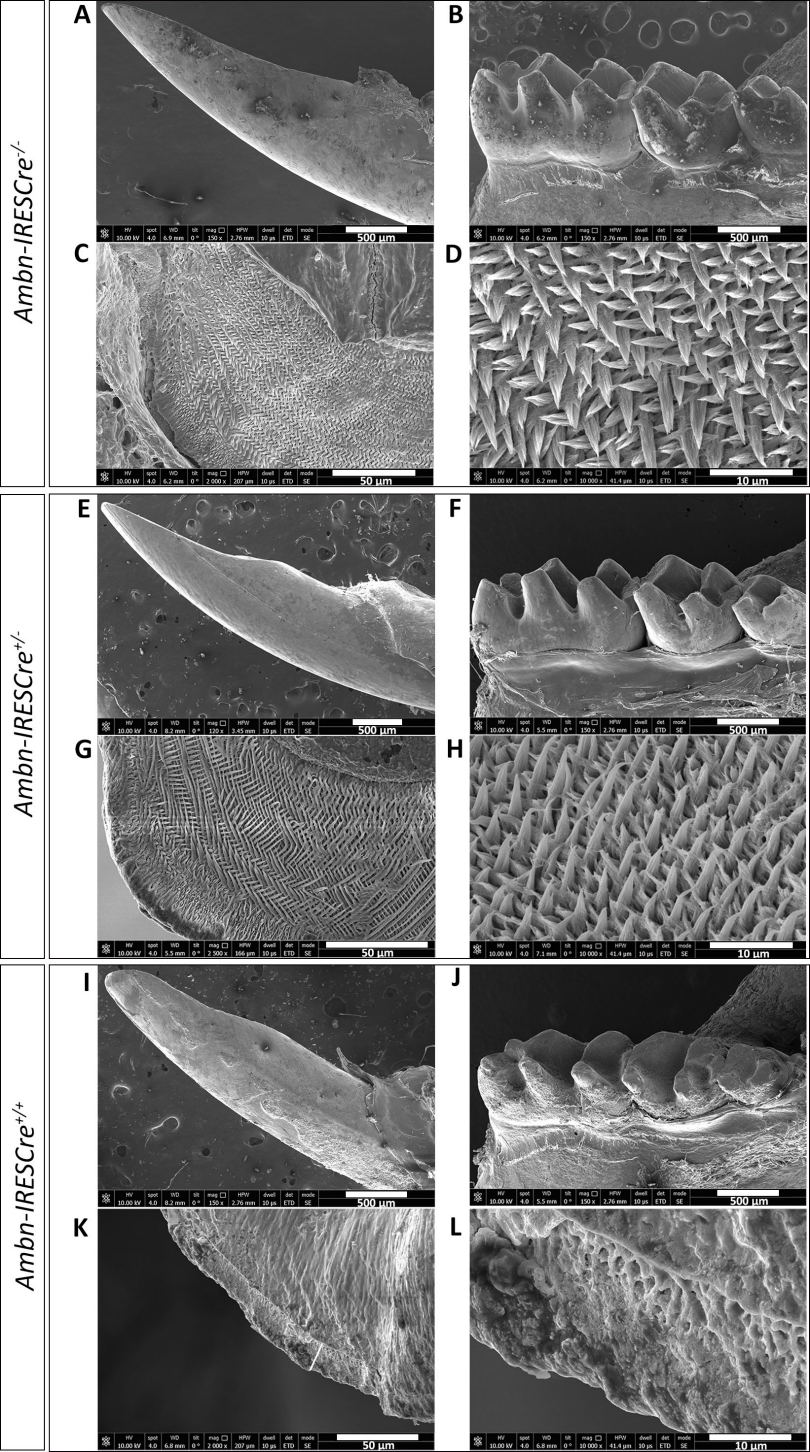
SEM images of 8-week-old *Ambn-IRESCre^-/-^* (WT), *Ambn-IRESCre^+/-^*, and *Ambn-IRESCre^+/+^* incisor enamel. The WT incisor **(A)** and molars **(B)** possessed a smooth, uniform layer of enamel, with the incisor tips and the molar cusp tips appearing sharp. The incisor enamel cross-section showed normal thickness and rod–interrod structure in WT **(C, D)**. The *Ambn-IRESCre^+/-^* incisor and molars were also smooth and sharp, the same as WT **(E, F)**. Cross-sections through the *Ambn-IRESCre^+/-^* incisor showed typical thickness and rod–interrod architecture **(G, H)** similar to WT. The *Ambn-IRESCre^+/+^* incisor and molar surfaces appeared rough, and the incisor tip and molar cusps were blunted **(I, J)**. The *Ambn-IRESCre^+/+^* enamel layer was thin (18.77 µm) **(K)**, and rod–interrod structure **(L)** was missing in the cross-section.

The SEM analysis revealed the smooth enamel surfaces of incisors and molars, with sharp incisor and molar cusp tips as well as normal enamel thickness (~114–120 μm) and normal rod–interrod architecture in WT (**Figures 5A–D**) and *Ambn-IRESCre^+/-^* incisors (**Figures 5E–H**). The *Ambn-IRESCre^+/+^* incisor and molars appeared blunt and had rough enamel surfaces (**Figures 5I, J**). Only a thin layer of enamel (~18.7 μm) with no true rod–interrod structure was found in *Ambn-IRESCre^+/+^* mice (**Figures 5K, L**).

A further investigation of the *Ambn-IRESCre^+/+^* mandible with H&E staining showed a single large cyst on the mesial surface of the first molar crown (**Supplementary Figures 8A–C**, arrowheads in A and C). The incisor cervical loop, pre-secretory, and secretory ameloblasts did not show gross abnormalities in H&E staining and secreted extracellular matrix (**Supplementary Figures 8D–F**), but from the transition stage onward, the ameloblasts were clearly dysmorphic, and pools of amorphous material could be observed within the ameloblast layer (**Supplementary Figures 8G–J**, arrow in H). Immunofluorescence revealed that the WT, *Ambn-IRESCre^+/-^*, and *Ambn-IRESCre^+/+^* incisors and molars expressed amelogenin and ameloblastin proteins (**Supplementary Figures 9**, **10**). Protein expression appeared unchanged between WT and *Ambn-IRESCre^+/-^* ameloblasts (**Supplementary Figure 9** and **S10A–H**), but in *Ambn-IRESCre^+/+^* incisor ameloblasts, less Ambn appeared in the extracellular matrix and more within the cells compared to WT and *Ambn-IRESCre^+/-^* ameloblasts (**Supplementary Figure 9** and **S10I–L**).

### *Ambn-IRESCre* ameloblast synthesizes both *Ambn* transcripts at a normal ratio

**Figure 6:**
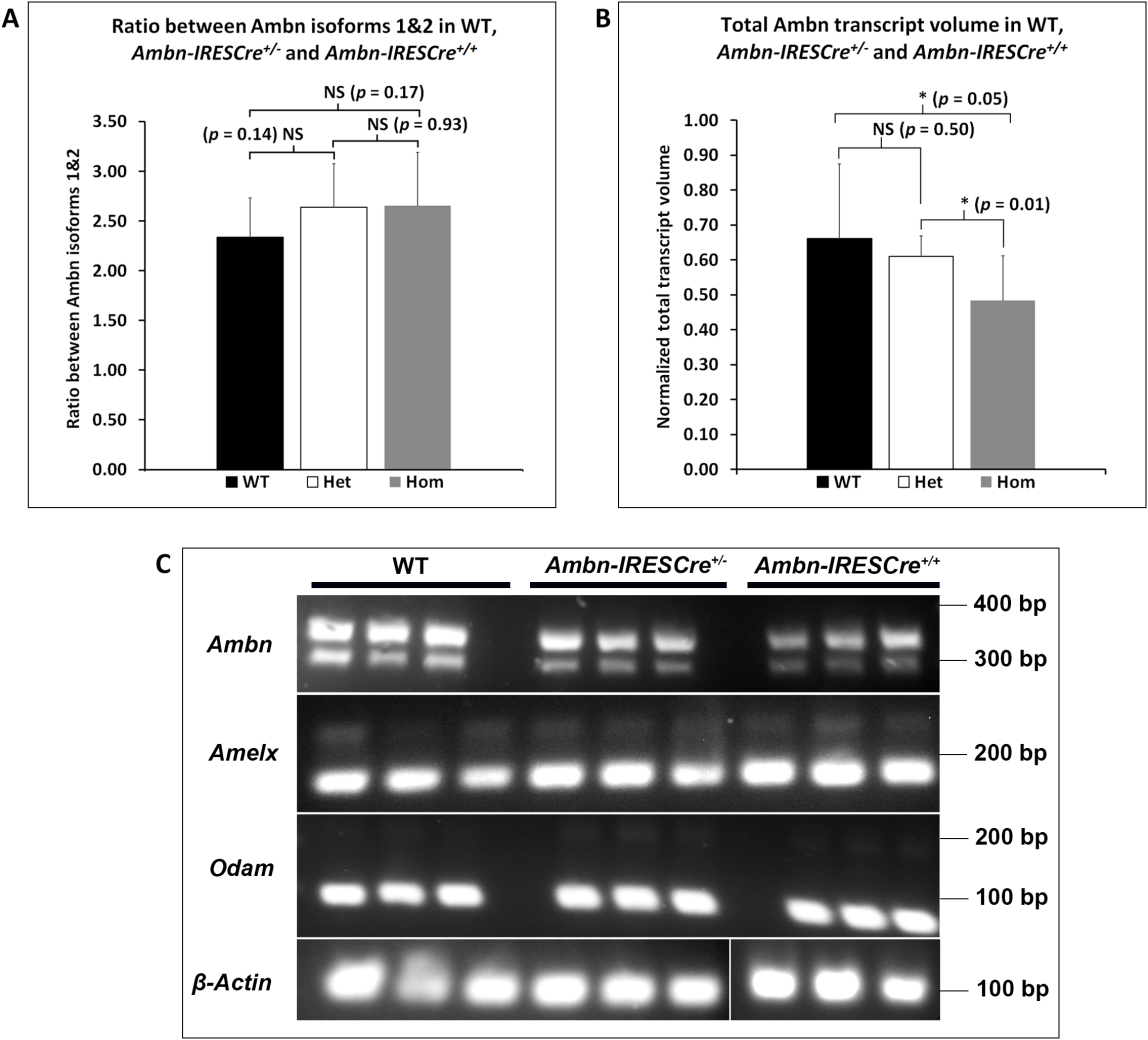
*Ambn* transcript analysis in PN14 WT, *Ambn-IRESCre^+/-^*, and *Ambn-IRESCre^+/+^* first molar enamel organs. No statistically significant differences (*p* > 0.05) were observed in the ratio between the two transcripts of *Ambn*, i.e., ratio of isoform 1 to isoform 2 band volumes in WT, *Ambn-IRESCre^+/-^*, and *Ambn-IRESCre^+/+^* first molar enamel organs **(A)**. The total volume of both *Ambn* isoforms together was significantly lower in *Ambn-IRESCre^+/+^* than *Ambn-IRESCre*^+/-^ and WT **(B)**. Agarose gel image showing the PCR products for *Ambn*, *Amelx*, and *Odam* transcript analysis, with *β-actin* used as control **(C)**. No differences were observed in the volumes of *Amelx* and *Odam* transcripts in the *Ambn-IRESCre^+/-^* and *Ambn-IRESCre^+/+^* animals compared to WT. Statistical analysis was performed using unpaired two-sample *t*-test, assuming unequal variances; *n* = 3 mice; *p ≤ 0.05.

To validate that the insertion of *Cre* in the 3′ UTR of *Ambn* did not affect its alternative splicing, we quantified the ratio and total volumes of two *Ambn* isoforms in WT, *Ambn-IRESCre^+/-^*, and *Ambn-IRESCre^+/+^* first molar enamel organs (**Figure 6**). The ratio between *Ambn* isoform 1 and isoform 2 remained unaltered in heterozygous and homozygous *Cre* animals (**Figures 6A, C**). However, the combined volume of both *Ambn* isoforms in *Ambn-IRESCre^+/+^* molars was significantly lower than their WT and *Ambn-IRESCre^+/-^* counterparts (**Figures 6B, C**). The *Ambn* isoforms’ volume in *Ambn-IRESCre^+/-^* was slightly lower than that of WT but not statistically different (**Figures 6B, C**). The *Amelx* and *Odam* transcripts were also visualized in a similar manner, and no differences were observed in their expression volume (**Figure 6C**).

### Eight-week-old *Ambn-IRESCre^+/-^/Smad4^fl/fl^* mice have a gross destructive enamel phenotype but are otherwise healthy

To determine the efficacy of the heretofore described *Ambn-IRESCre* mouse line, we used the heterozygous Cre animals to generate a Cre/*loxP* mutant. We crossed *Smad4^fl/fl^* mice with *Ambn-IRESCre^+/-^* to produce *Smad4* conditional knockout *Ambn-IRESCre^+/^/Smad4^fl/fl^*, simply referred to as mutants.

**Figure 7:**
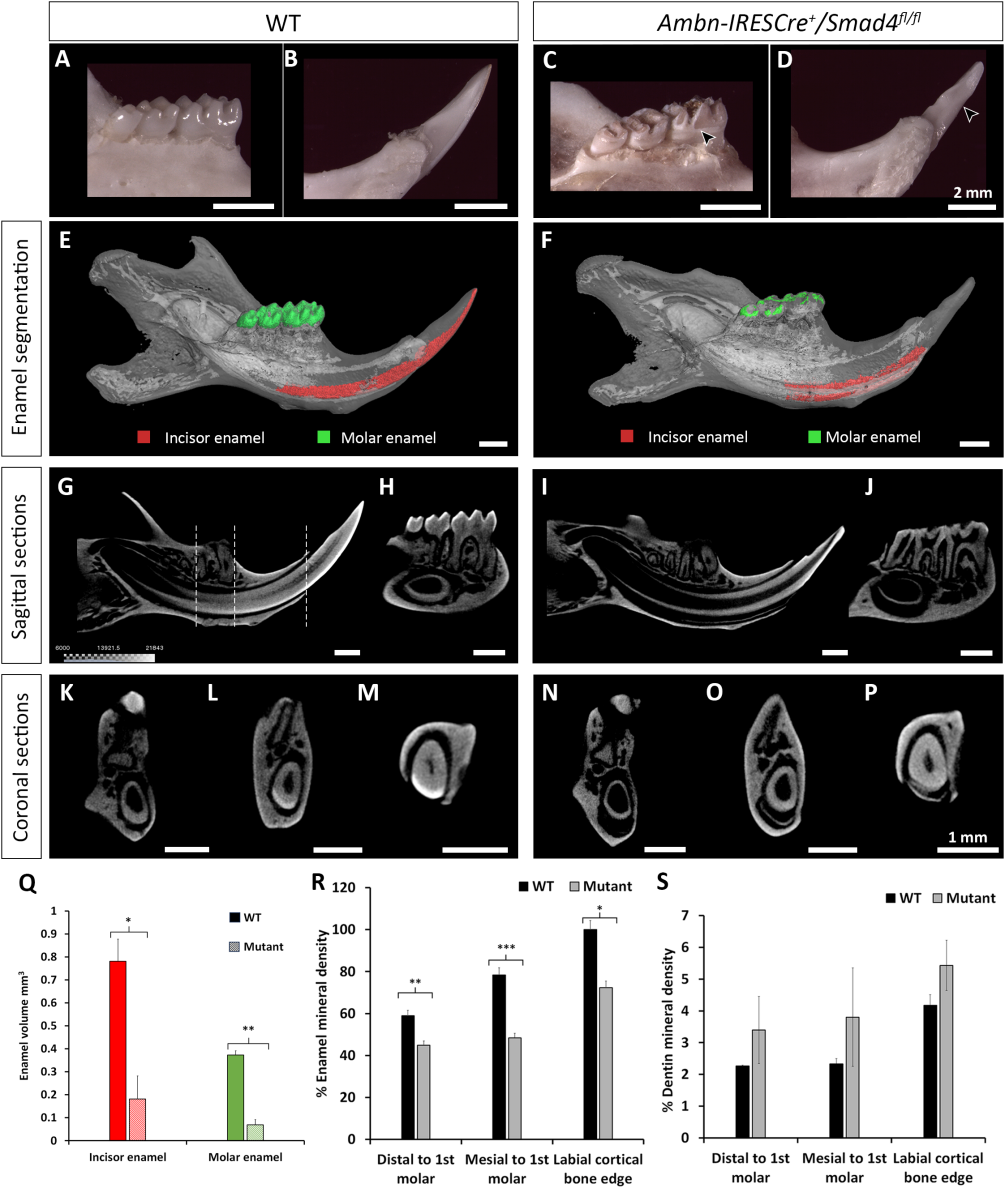
Photographic images and μCT analysis of 8-week-old WT and *Ambn-IRESCre^+/-^/Smad4^fl/fl^* mutant mouse molars and incisor. The WT enamel appears smooth and translucent on the surface of molars and incisors **(A, B)**. Mutant molars and incisor show severe enamel loss, arrowheads in **(C, D)**. Three-dimensional μCT reconstructions of WT **(E)** and *Ambn-IRESCre^+/-^/Smad4^fl/fl^* **(F)** mandibles with incisor enamel pseudo-colored in red and molar enamel in green, further demonstrating attrition in the mutants. Sagittal sections from μCT reconstructions of WT incisor and molars in **(G, H)** and *Ambn-IRESCre^+/-^/Smad4^fl/fl^* in **(I, J)**. The mutant sagittal section shows short, thin, and blunted incisor **(I)** and molars lacking sharply defined cusps **(J)** due to loss of enamel. Coronal μCT sections **(K–M)** of the WT incisor taken at three regions shown in **(G)** by dotted lines: distal to the distal cusp of the first molar **(K)**, mesial to the mesial cusp of the first molar **(L)**, and labial cortical bone edge **(M)** that shows uniform, mineralized enamel in WT. The *Ambn-IRESCre^+/-^/Smad4^fl/fl^* μCT sections taken at the same regions show severely hypomineralized enamel **(N–P)**. Grayscale values for **(G–P)** are shown in **(G)**; scale for **(G–P)** is shown in **(P)**. Graphs representing mean values with standard deviation of enamel volume **(Q)**, percent relative enamel mineral density **(R)**, and percent relative dentin mineral density **(S)**. The incisor and molar enamel volumes **(Q)** in WT were 0.78 and 0.37 mm^3^ (quantified from the red and green voxels in **(E)**, respectively), while for the *Ambn-IRESCre^+/-^/Smad4^fl/fl^* mice these were 0.18 and 0.06 mm^3^ (quantified from the red and green voxels in **(F)**, respectively). The mutant enamel volumes were significantly lower than those of WT **(Q)**. The mineral density of *Ambn-IRESCre^+/-^/Smad4^fl/fl^* incisor enamel measured distal to the first molar was 44%, mesial to the first molar was 48%, and at the labial cortical bone edge was 72% that of WT, which was significantly lower than WT in all three regions **(R)**. There were no significant differences observed in the mineral density of mutant and WT dentin **(S)**. Statistical analysis was performed using unpaired two-sample *t*-test assuming unequal variances; *n* = 6 mice; *p ≤ 0.05, **p ≤ 0.01, ***p ≤ 0.001.

The mandibular incisors and molars of 8-week-old *Ambn-IRESCre^+/-^/Smad4^fl/fl^* mutants were examined using µCT, SEM, and BS-SEM. Apart from the dental phenotype, the *Ambn-IRESCre^+/-^/Smad4^fl/fl^* mice were otherwise healthy and showed normal breeding patterns. They were of normal size and weight when euthanized. At 8 weeks, the mutant enamel was hypomineralized and post-eruption had extensive regions of fracture and loss of enamel (**Figure 7**; **Supplementary Figure 11**). While the WT enamel appeared smooth and translucent (**Figures 7A, B**), the *Ambn-IRESCre^+/-^/Smad4^fl/fl^* teeth showed only isolated regions of opaque white enamel (**Figures 7C, D**). Pulpal exposures on some of the mandibular incisors were also noted (**Supplementary Figure 11C**), while on the molars, the occlusal enamel was generally worn or fractured away, leaving behind ridges of enamel on the cervical or buccal thirds (**Figure 7C**, arrowhead, **Figures 7F, J**; **Supplementary Figure 11D**).

Based on the μCT data, enamel segmentation analysis was performed using three-dimensional reconstructions to quantify the loss of enamel in *Ambn-IRESCre^+/-^/Smad4^fl/fl^* mutant mice (**Figures 7E, F, Q**). There was greater than fourfold loss in mutant incisor enamel volume compared to that of the wild-type incisor and greater than sixfold loss of mutant molar enamel volume compared to that of wild-type molars (**Figure 7Q**). When quantifying mutant enamel mineral density, areas of intact enamel were carefully selected at the previously chosen anatomical landmarks (**Figure 7G**, dotted lines). Mutant incisor enamel was significantly hypomineralized at all three regions compared to wild type (**Figures 7K–P, R**). Since the pulp chambers in the mutant incisors appeared larger than those in WT (particularly **Figure 7N**), the incisor dentin surface area was measured in the μCT coronal sections at the abovementioned three anatomical landmarks. It was significantly lower at the distal to the first molar region, making the pulp chamber in the mutant incisors appear larger. It was not significantly different from WT at the mesial to the first molar and at the labial cortical bone edge (**Supplementary Figure 12A**). There were no significant changes in dentin mineral density in the incisors (**Figure 7S**) or molars (**Supplementary Figure 12B**) as well as the total volume of incisor dentin (**Supplementary Figure 13**) in *Ambn-IRESCre^+/-^/Smad4^fl/fl^* mice. The *Ambn-IRESCre^+/-^/Smad4^fl/fl^* odontoblasts as well as the root structure appeared normal except for the presence of keratin pearls near the molar roots (**Supplementary Figure 14**).

**Figure 8:**
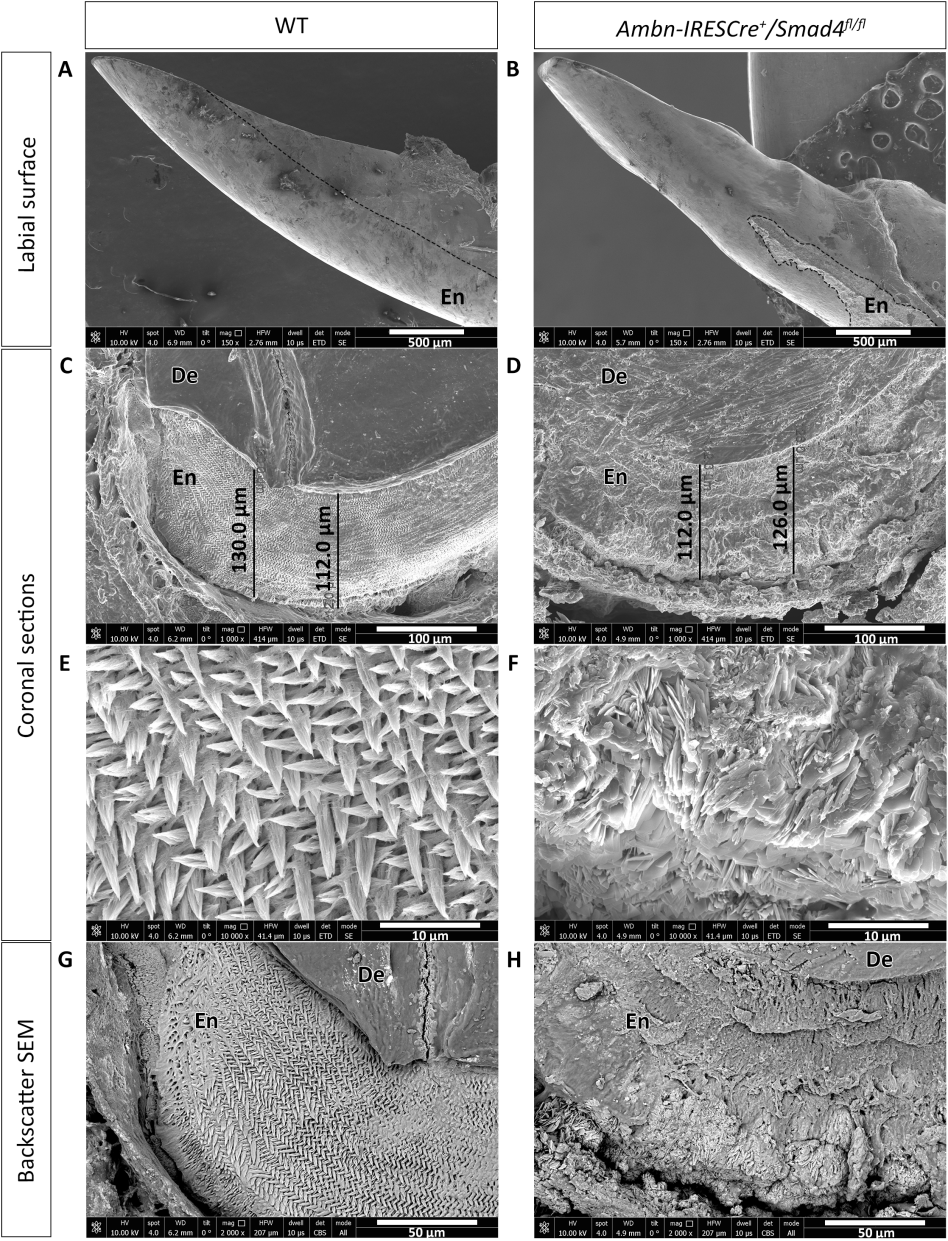
SEM and BS-SEM images of WT and *Ambn-IRESCre^+/-^/Smad4^fl/fl^* mutants. The labial surface of the WT incisor **(A)** was completely covered in enamel (the dotted line indicates dentin enamel junction), while the mutant incisor **(B)** showed scant patches of enamel [dotted outline in **(B)**]. The thickness of the WT enamel prior to eruption was between 112 and 130 μm **(C)**, which was similar to that of unerupted mutant enamel [112–126 μm, **(D)**]. The WT enamel possessed well organized rod–interrod architecture **(E)** which was lacking in the mutant enamel, which instead had large, flat, plate-like crystals that were haphazardly arranged **(F)**. The WT enamel was distinctly more mineralized than dentin **(G)**, but the hypomineralized mutant enamel appeared similar in brightness to dentin in the BS-SEM images **(H)**. En, enamel; De, dentin.

We then sought to determine if any enamel prismatic structure was evident in the *Ambn-IRESCre^+/-^/Smad4^fl/fl^* mice (**Figure 8**). The WT incisor had a smooth continuous layer of enamel on its labial surface (**Figure 8A**, En). Only scant areas of enamel were visible on the labial surface of the *Ambn-IRESCre^+/-^/Smad4^fl/fl^* incisor (**Figure 8B**, En). Using SEM and BS-SEM focused on intact incisor enamel just prior to eruption (distal to the labial cortical bone edge), we determined that the thickness of the mutant enamel before being delaminated was similar to that of WT (**Figures 8C, D**). Upon studying the prismatic architecture, we encountered some technical challenges. Acid etching the cut surface of the incisors prior to SEM was essential to remove the smear layer, but the mutant enamel was rapidly destroyed upon acid etching, presumably because of extreme hypomineralization as evident in the µCT data (shown in **Figure 7**). Very short etching (~10 s as opposed to 40–45 s for WT) of the mutant samples revealed an absence of decussating rod–interrod structure characteristic of murine enamel (**Figure 8F**). The WT enamel was composed of well-organized thin and needle-like crystals bundled together in enamel rods (**Figure 8E**), whereas disorganized, large, flat, and platelike crystals were seen in the mutant (**Figure 8F**). The wild-type enamel was more mineralized than dentin and hence appeared brighter in BS-SEM images (**Figure 8G**). The BS-SEM confirmed the hypomineralization of mutant enamel which appeared similar in brightness (representing mineral content) to dentin (**Figure 8H**).

### Eight-week-old *Ambn-IRESCre^+/-^/Smad4^fl/fl^* ameloblasts are severely pathological

**Figure 9:**
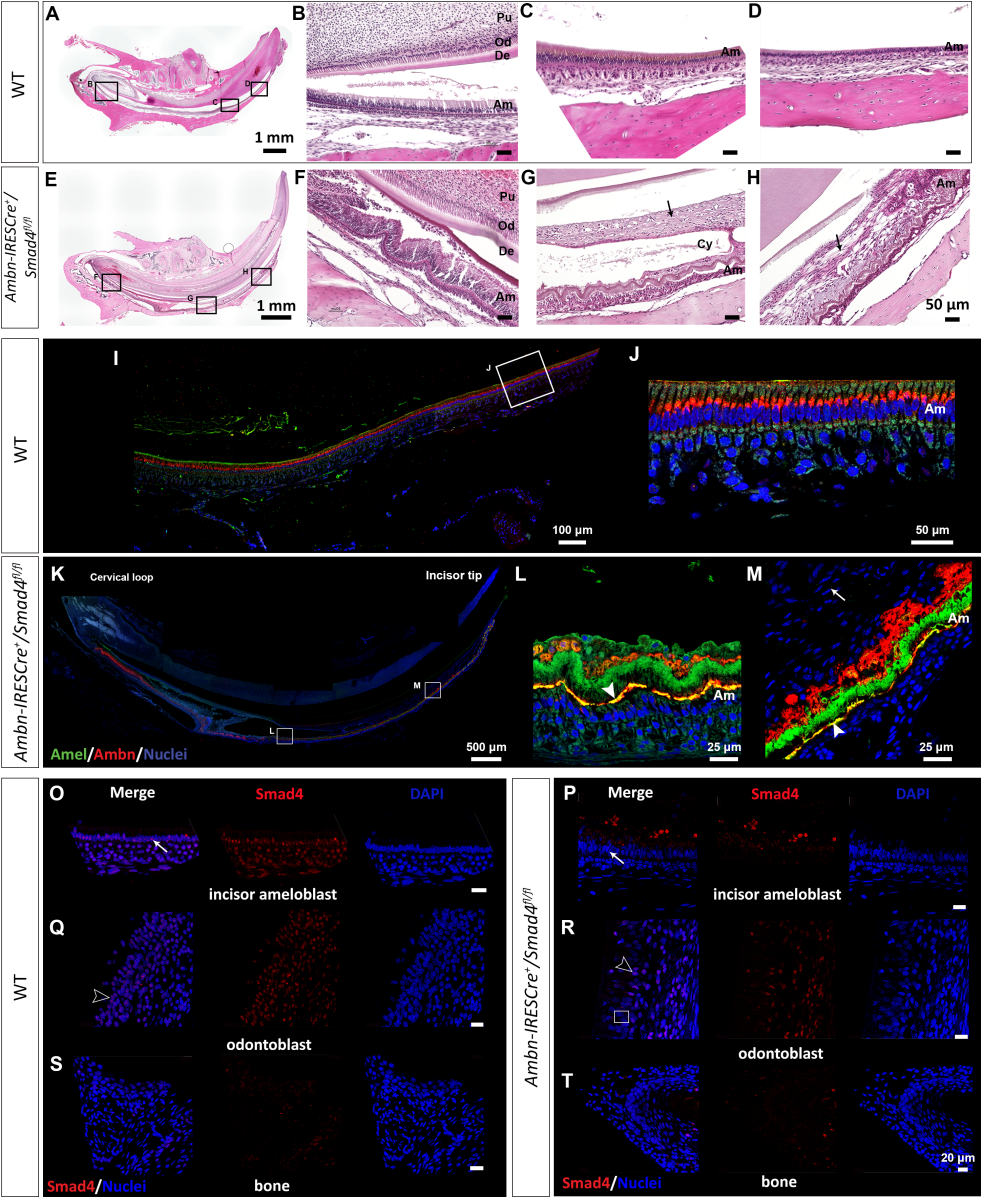
Brightfield microscopic images of WT and *Ambn-IRESCre^+/-^/Smad4^fl/fl^* mandibles stained with hematoxylin and eosin. WT ameloblasts appeared in a single layer of tall columnar cells with basal nuclei **(A–D)**. **(B–D)** Secretory, transition, and maturation stages. *Ambn-IRESCre^+/-^/Smad4^fl/fl^* **(E**–**H)** secretory stage ameloblasts appeared scalloped **(F)**. A cyst (Cy) was visible in the late secretory/transition stage **(G)** region lined by a thick layer of spindle-shaped cells [**(G)**, arrow] toward the dentin side and by ameloblasts toward the base. The layer of spindle-shaped cells continued apical to the cyst in the maturation stage **(H)** of the incisor [**(H)**, arrow]. Confocal microscopic images of WT **(I, J)** and *Ambn-IRESCre^+/-^/Smad4^fl/fl^* incisor **(K–M)** immunolabeled for amelogenin (green) and ameloblastin (red) with the nuclei stained with DAPI (blue). Amelogenin and ameloblastin colocalized at the apical end of ameloblasts in both WT **(J)** and mutants [**(L)**, arrowhead]. Both proteins also pooled within the mutant enamel matrix, appearing as distinct layers of green and red **(L)**. At maturation stage, discrete deposits of both amelogenin and ameloblastin continued to be visible apical to the ameloblasts **(M)**, along with a thin line of colocalized proteins [**(M)**, arrowhead]. The infiltrate of spindle-shaped cells did not express amelogenin or ameloblastin [**(M)**, arrow]. Am, ameloblasts; De, dentin; Od, odontoblasts; Pu, pulp; Cy, cyst. Confocal microscopic images of WT **(O, Q, S)** and *Ambn-IRESCre^+/-^/Smad4^fl/fl^* **(P, R, T)** incisor ameloblast, odontoblast, and bone, respectively, immunolabeled for Smad4 protein in red. The WT incisor ameloblast showed consistent nuclear Smad4 labeling [**(O)**, arrow]. The mutant incisor ameloblast lacked nuclear localization of Smad4 [**(P)**, arrow] but showed supranuclear Smad4 labeling along with some non-specific red labeling outside the ameloblast. WT and mutant odontoblast (arrowheads in **(Q, R)**, respectively) and pulp cells were also positive for Smad4 immunolabeled in the nucleus, but few mutant odontoblast lacked Smad4 labeling [box in **(R)**]. Smad4 detection in bone was weak in both WT and mutants [**(S, T)**, respectively], and similar sparse red punctate labeling was observed in the nuclei of both samples.

The 8-week-old WT incisor ameloblasts exhibited a characteristically tall, columnar, and single-layer appearance (**Figures 9A, D**). The *Ambn-IRESCre^+/-^/Smad4^fl/fl^* ameloblasts were multilayered and scalloped (**Figures 9E–H**). Cysts developed within the ameloblast layer in severe cases (**Figure 9G**). At the transition and maturation stages, a thick layer of spindle-like cells appeared apical to the ameloblasts (**Figures 9G, H**, arrows). The thickness of this layer varied between each mutant sample examined (**Supplementary Figures 15A–C**). Pathological ameloblasts were observed near the cervical loop region at the early secretory stage in the mutants (**Supplementary Figures 15D, E**). Cytokeratin-14 immunofluorescence labeled both WT and mutant ameloblast (**Supplementary Figures 16A–D**) and keratin pearls near the roots of first and second molars (**Supplementary Figure 16C**, arrow), but the spindle-like cells infiltrating the enamel organ were not positive for cytokeratin-14 (**Supplementary Figure 16D**, arrowhead).

### Residual Amel and Ambn proteins are observed in mutant enamel matrix

Amelogenin (Amel) and ameloblastin (Ambn) co-immunofluorescence was used to detect the enamel matrix proteins in WT and mutant ameloblasts (**Figure 9**). The proteins colocalized at the apical end of ameloblasts in both WT (**Figures 9I, J**) and mutants (**Figures 9K–M**, arrowheads in L and M). Unlike WT ameloblasts, in which amelogenin and ameloblastin appeared in isolation only intracellularly (**Figure 9J**), discrete layers of amelogenin (green) and ameloblastin (red) were observed in the extracellular matrix apical to the incisor ameloblasts (**Figures 9L, M**) in the mutants. These layers were further covered by the spindle-like cells which were negative for both Amel and Ambn proteins (**Figure 9M**, arrow). This suggested that although the mutant ameloblasts secreted Amel and Ambn proteins, their digestion, assembly, and/or re-uptake may have been disrupted.

### Smad4 nuclear localization is inhibited in mutant ameloblast

Smad4 immunofluorescence revealed distinct nuclear labeling in WT ameloblast (**Figure 9O**, arrow). However, similar labeling was absent in mutants (**Figure 9P**, arrow), with Smad4 being seen supranuclearly in mutant ameloblast instead. In both WT and mutant odontoblast, a distinct Smad4 signal within the nuclei could be observed (**Figures 9Q, R**, arrowheads). Consistent with the Cre-recombination pattern seen in *Ambn-IRESCre/mTmG* mice, not all mutant odontoblast were positive for Smad4 (**Figure 9R**. box). Although the antibody used in this study did not strongly detect Smad4 in WT bone (**Figure 9S**), similar sparse nuclear labeling was also detected in mutant bone (**Figure 9T**).

### *Ambn-IRESCre^+^/Smad4^fl/fl^* ameloblasts have downregulated *Ambn* and *Odam*

We analyzed the expression of *Amelx*, *Ambn*, *Odam*, *Smad4*, and *Cre* in *Ambn-IRESCre^+/-^/Smad4^fl/fl^* PN14 first molar enamel organs by q-RTPCR (**Supplementary Figure 17**). Sample to sample variability was observed due to the severe pathology present in ameloblasts. Overall, *Smad4* was downregulated anywhere between -1.4 and -6.7-fold (average, -2.2-fold). *Ambn* and *Odam* were also downregulated in all samples, while *Amelx* remained unaffected in two out of three samples analyzed.

These data suggest that Smad4 activity is essential during multiple stages of amelogenesis, prior to and during secretory stage, but well beyond the dental bud stage where pre-odontoblasts and pre-ameloblasts are being established. Furthermore, we conclude that the *Ambn-IRESCre^+/-^* driver mouse line is working as intended in creating predominantly ameloblast-specific gene knockouts.

## Discussion

We have generated a new *Ambn-IRESCre* mouse line to study gene activities that define amelogenesis. We examined the RNA expression of *Ambn* and *Cre* by ISH and observed the spatiotemporal expression of Cre-recombinase in *Ambn-IRESCre* mice using two reporter mouse lines. We also closely examined both *Ambn-IRESCre^+/-^* and *Ambn-IRESCre^+/+^* enamel phenotypes at the macroscopic (micro-computed tomography) and microscopic (SEM) levels to determine if this mouse model is suitable for generating new Cre–*loxP* mutations. To further showcase the value of this novel *Ambn-IRESCre* mouse line, we generated an *Ambn-IRESCre^+/-^/Smad4^fl/fl^* mutant.

There were no adverse effects observed from a single allele of *Cre*-recombinase, and the *Ambn-IRESCre*^+/-^ enamel was normal, with the mineral density and the rod–interrod structure similar to WT.

To directly examine the spatiotemporal expression of *Ambn* and *Cre* RNA, we utilized *in situ* hybridization (ISH). The *Ambn* expression pattern closely mimicked prior studies (Bosshardt and Nanci, 1998; Fong et al., 1998; Krebsbach et al., 1996), initiating in pre-ameloblast and pre-odontoblast and continuing robustly in ameloblast while being absent in mature odontoblast. The *Cre* RNA expression matched that of *Ambn*, with the exception of pulp, pre-odontoblast, and late-maturation-stage ameloblast. In these three cell types, the *Cre* expression was lower compared to that of *Ambn*, i.e., not all pulp cells, pre-odontoblasts, and maturation-stage ameloblasts that expressed *Ambn* also expressed *Cre* RNA. Therefore, to directly visualize the effects of Cre-recombination, we utilized mTmG reporter mice. Due to FFPE processing of the tissue, anti-GFP antibody was used to amplify the GFP signal. Cre-dependent mTmG recombination was most robust in ameloblast from secretory to maturation stage in both incisors and molars. Although *Ambn* and *Cre* RNA were detected in subsets of dental mesenchymal cells, only a few odontoblast and pulp cells showed green fluorescence, possibly as a consequence of *Ambn* expression being lower in these compared to ameloblast (Jacques et al., 2014). Intermittent GFP signal was also observed in the alveolar bone consistent with previously published *Ambn* expression profile (Jacques et al., 2014).

We then utilized the R26R-LacZ reporter mouse line for its high sensitivity and ease of detection (Krämer et al., 2021) to examine the jaws of *Ambn-IRESCre^+^/R26R^+^* mice at three different timepoints (post-natal-days 3, 5, and 8). LacZ staining started immediately in early secretory ameloblasts and was seen in pulp and pre-odontoblasts, along with incisor and molar ameloblasts until maturation stage. Unlike in *Ambn-IRESCre/mTmG*, the mature odontoblasts were positive for LacZ due to permanent lineage marking in the R26R strain (Srinivas et al., 2001; Lan et al., 2007).

Based on the findings from the two reporter lines, *Ambn-IRESCre* activity is expected to follow endogenous *Ambn* expression with robust Cre expression in ameloblast along with partial/mosaic recombination in odontoblast-lineage/pulp cells and alveolar bone (Jacques et al., 2014; Spoutil et al., 2023). Lastly, *Ambn-IRESCre*^+/-^ ameloblasts normally expressed both isoforms of *Ambn* RNA and the ameloblastin protein, confirming that the insertion of a single Cre allele in the 3′ UTR region of *Ambn* did not affect its alternative splicing or its protein expression. We did not examine the embryonic stages prior to 3 days post-birth; therefore, the precise onset of Cre activity during early molar morphogenesis cannot be determined from the present study.

The *Ambn-IRESCre^+/+^* mice showed a destructive phenotype limited to ameloblasts. Given that the 3′ UTR regions in both *Ambn* alleles were replaced by Cre, an unexpected *Ambn* loss-of-function was suspected (Mayr, 2019). However, the phenotype in the *Ambn-IRESCre^+/+^* mice differed from *Ambn* KO (Liang et al., 2019) in three ways, namely: (1) *Ambn-IRESCre^+/+^* ameloblasts expressed both isoforms of ameloblastin RNA in similar ratios to WT and *Ambn-IRESCre^+/-^* but with a reduced RNA amount, (2) *Ambn-IRESCre^+/+^* ameloblasts also expressed ameloblastin protein as confirmed by immunofluorescence, and (3) *Ambn-IRESCre^+/+^* mice secreted enamel extracellular matrix and formed a thin layer of enamel on the surface of dentin as observed by H&E staining and electron microscopy. Such a layer was absent in *Ambn* KO mice which only formed abnormal calcified nodules on the dentin surface (Liang et al., 2019). Although we did not further investigate the causes behind this phenotype, reduced quantities of Ambn could be one of the factors.

Based on this validation, we state that the spatiotemporal expression of *Cre* in *Ambn-IRESCre^+/-^* mice was as intended with no adverse phenotypes and recommend that only heterozygous *Ambn-IRESCre* mice be used to generate Cre/*loxP* crosses to study gene functions in ameloblasts.

Using the heterozygous *Ambn-IRESCre* mice, we generated *Ambn-IRESCre^+/-^/Smad4^fl/fl^* conditional knockout mice to demonstrate the capabilities of the new *Cre* driver line. The *Krt14-Cre^+^/Smad4^fl/fl^* mice studied previously presented major disruptions to the craniofacial region as early as embryonic day 11, and these mice died at birth (Huang et al., 2010; Ko et al., 2007). Major craniofacial developmental pathologies and early mortality meant that any *in vivo* interpretations of the specific contributions of Smad4 to amelogenesis beyond the pre-secretory stage were not possible. Our *Ambn-IRESCre^+/-^/Smad4^fl/fl^* mice were viable at birth with no life-threatening phenotypes post-birth. The conditional knockdown of *Smad4* under *Ambn* driven *Cre* resulted in significant and specific disruption of amelogenesis, while the mice remained otherwise healthy. Smad4 immunofluorescence detected a lack of nuclear Smad4 labeling in mutant ameloblasts.

At PN14, there was significant downregulation of *Smad4* mRNA in the molar enamel organs, albeit with variation between individual samples. Smad4 immunolabeling in ameloblasts indicated that the Smad4 protein was not completely absent; however, it failed to localize efficiently to the nucleus, suggesting a loss of functional Smad4 signaling rather than complete elimination of all detectable Smad4 protein (Liu et al., 1997). This would suggest that recombined mutant ameloblasts continue to produce a truncated *Smad4* transcript, which may account for the residual *Smad4* mRNA detected by qPCR. In addition, non-ameloblast tissues of the enamel organ which are inevitably isolated along with ameloblasts, as well as in the infiltrate of unidentified spindle-shaped cells in the mutants, may also contribute to the variable *Smad4* gene expression.

At 8 weeks, the *Ambn-IRESCre^+/-^/Smad4^fl/fl^* dentition had a gross destructive enamel phenotype. Besides enamel, the dentin had a mild phenotype in the early stages of development distal to the first molar, which recovered anterior to the first molar, and there were no overall deleterious effects on incisor dentin volume or dentin mineral density. This seems reasonable given that immunofluorescence revealed nuclear localization of Smad4 in majority of the mature odontoblasts, while only a few lacked nuclear Smad4 signals, matching the recombination pattern observed in *Ambn-IRESCre^+/-^/mTmG^+/-^*.

It is important to note that in the 8-week-old *Ambn-IRESCre^+/-^/Smad4^fl/fl^* mice, most of the enamel was fractured or completely lost post-eruption. Upon SEM imaging, we observed large, flat, plate-like crystals instead of a regular prismatic organization in the mutant enamel, but the thickness of unerupted enamel was unaltered. While pulpal exposures were not evident in any of the 8-week *Ambn-IRESCre^+/-^/Smad4^fl/fl^* molars, pulpal exposures were noted in the mutant incisors. Even though mutant incisor ameloblasts displayed abnormal morphology from the pre-ameloblast stage, they secreted both amelogenin and ameloblastin enamel matrix proteins. The infiltration of spindle-like cells within the enamel organ was also a novel finding in this mutant. Canonical BMP/TGF-β signaling through Smad4 is essential for epithelial differentiation and tissue organization during tooth development (Huang et al., 2010; Xu et al., 2008). The early deletion of *Smad4* using *Keratin-14Cre* leads to enamel patterning defects but does not directly inhibit ameloblast differentiation and amelogenin expression (Huang et al., 2010). These published results support our finding that Smad4 plays crucial roles throughout all stages of enamel formation, thus maintaining the overall integrity of the enamel organ and contributing to ameloblast morphology, ameloblast movement, and possibly the digestion and re-uptake of enamel matrix proteins, but it may be dispensable for enamel matrix protein secretion. Although it is out of the scope of this study to investigate the downstream effects of Smad4 deletion within ameloblasts, the *Ambn-IRESCre^+/-^/Smad4^fl/fl^* mouse model uniquely allows the interrelation of these pathways to be studied in the future.

Our data confirmed that the use of IRESCre cassette inserted directly after the targeted *Ambn* gene stop codon allows for the expression of Cre under the control of the endogenous *Ambn* promoter. Collectively, the development of the *Ambn-IRESCre* (catalog # 067446-JAX; C57BL/6J-*Ambn^em1Mlp^*/Mmjax) mouse line provides a powerful tool for conditional knockout studies focused on amelogenesis.

## Data Availability

The datasets presented in this study can be found in online repositories. The data have been uploaded to FaceBase repository. Ambn-IRESCre data: Accession #FB00001404. Link: https://www.facebase.org/id/7X-HZDA. Ambn-IRESCre/Smad4 mutant data: Accession #FB00001355. Link: https://www.facebase.org/id/2W-YN1C.
