# Supplementary material for: A novel Ambn-IRESCre mouse line allows ameloblast-specific Smad4 silencing
Source: Front Physiol. 2026 Jun 3;17:1792453. doi: 10.3389/fphys.2026.1792453 (PMC13272140; doi:10.3389/fphys.2026.1792453)
Supplement: Supplementary file 1 [file DataSheet1.pdf]

A Novel *Ambn-IRESCre* Mouse Line Allows Ameloblast Specific *Smad4* Silencing

Supplementary Material

Rucha Arun Bapat[1], Yanbin Ji[1], Marziyeh Aghazadeh[1], Alexis E. Bauer[1], David C. Evans[1], Joseph G. Hacia[2], Yan Zhou[1,3] and Michael L. Paine[1,3,*]

[1] Center for Craniofacial Molecular Biology, Herman Ostrow School of Dentistry of the University of Southern California, Los Angeles CA 90033

[2] Department of Cancer Biology, Keck School of Medicine of the University of Southern California, Los Angeles CA 90033

[3] Department of Biomedical Sciences, Herman Ostrow School of Dentistry of the University of Southern California, Los Angeles CA 90033

*** Corresponding author:**

Michael L. Paine

Center for Craniofacial Molecular Biology
Department of Biomedical Sciences
Herman Ostrow School of Dentistry
University of Southern California
2250 Alcazar Street, CSA103, Los Angeles, CA 90033, USA
e-mail: paine@usc.edu

phone: 323-442-1728

Keywords: ameloblast, ameloblastin, IRES-Cre, enamel, Smad4 knockout mice

Short Title: *Ambn-IRESCre* Mouse Line

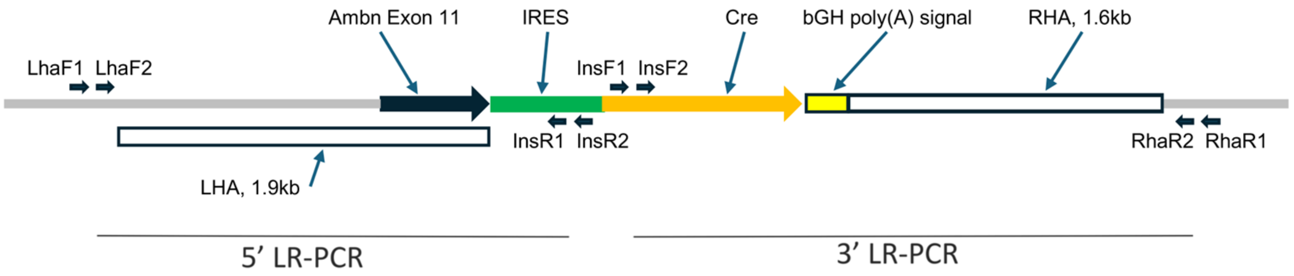

5′ LR-PCR; 2.4kb
LhaF1: GTGGTGATGCACAGAATCGC
LhaF2: CTCAGGATGTGGAGGCAAGA
InsR1: ACGCTTGAGGAGAGCCATTT
InsR2: GCATCCTTCAGCCCCTTGTT

3′ LR-PCR; 2.8kb
InsF1: CCCGCAGAACCTGAAGATGT
InsF2: CGCGGTCTGGCAGTAAAAAC
RhaR1: CCCAACATTAAGCCTGGGGT
RhaR2: TGGAGCAGCACTGTTACAGG

Supplementary Figure 1. Schematic of the development of the *Ambn-IRESCre* mouse and sequencing strategy. CRISPR/Cas9 technology was used to insert the “IRES/Cre recombinase/bGH poly(A) signal” cassette, into the *Ambn* gene locus immediately following its stop codon. The PCR primers identified were used to amplify DNA, sequence, and confirm stable integration in both the founder mice (F1) and the first backcross generation (N1), and without evidence of random integration event. IRES: Internal Ribosome Entry Site; RHA/Rha: right homology arm; LHA/Lha: left homology arm. Image from The Jackson Laboratory (JAX).

	Probe PCR				Products	Source
1a	Ambn-IRESCre	CGTTTCCAAGAGCCCTGA (f.c.)	CACACCGGCCTTATTCCAAG (r.m.)	CAAGGTACTATGGGGACAAAGC (r.WT)	74 bp (m.), 84 bp (WT)	
1b			CCCTAACGTTACTGGCCGA (p.m.)	TAACCTTGACATAGCAGCTACTTTGT (p.WT)		
2a	R26R LacZ	CTGGCTTCTGAGGACCG (f.c.)	CGAAGAGTTTGTCCTCAACCG (r.m.)	AATCTGTGGGAAGTCTTGTCC (r.WT)	195 bp (m.), 193 bp (WT)	JAX #003474
2b			ACCCTGGACTACTGCGCCC (p.m)	TAACCTGGTGTGTGGGCGTTGT (p.WT)		
	**Standard PCR**				**Products**	**Source**
1	Ambn-IRESCre	GGTGATGGAGAAGCAACCAT (f.c.)	ACACCGGCCTTATTCCAAG (r.m.)	TGGAAGCAAGAAGGGACCTA (r.WT)	190 bp (m.), 292 bp (WT)	MMRRC #67446
2	R26R LacZ	AAAGTCGCTCTGAGTTGTTAT (f.c.)	GCGAAGAGTTTGTCCTCAACC (r.m.)	GGAGCGGGAGAAATGGATATG (r.WT)	300 bp (m.), 603 bp (WT)	JAX #003474
3	Smad4 floxed	CAGAGTGGGTCTTTCTACCTTAGT (f.c.)	CAAGCTTTGAGAATGTCTGTGATAG (r.c.)		119 bp (m.), 65 bp (WT)	JAX #017462
4	mTmG	GCTAACCATGTTCATGCCTTC (f.m.)	CATGAACTCTTTGATGACCTC (r.m.)		280 bp (m.)	JAX #007676
			GTTCTCTGCTGCCTCCTG (f.WT)	CGGATCACAAGCAATAATAACC (r.WT)	320 bp (WT)	

***Supplementary Table 1**:* Primers 1a, 1b, 2a and 2b are for fluorescent probe PCR performed by Jackson laboratories. PCR primers used for genotyping *Ambn-IRESCre*, *R26R Smad4* floxed and mTmG mice are #1-4. f.c.: forward common; r.c.: reverse common; r.m: reverse mutant; r.WT: reverse wild type; p.m.: probe mutant; and p.WT: probe wild type.

Gene	Forward primer	Reverse primer	Source
AmelX	GGGGACCTGGATTTTG TTTG	AACCATAGGAAGGATACGGCT G	(Lacruz et al. 2012)
Ambn	GAGCCTTGAGACAATG AGACAG	GAGAAGTCCGTGCAACCATAA	
Ambn#	CTTCTCCCACCGCATAA CTCTT	TGGAACACGGTTGGAAATTGT G	
Odam	TGCTAGCCCTATGTCCT ATGT	CCAGGGCAGAAGCATGTAAA	
Smad4	CAGCCTCCCATTTCCAA TCATC	CGAAGGATCCACATAGCCATC C	(Sakai et al. 2019)
Cre	GCGGTCTGGCAGTAAA AACTATC	GTGAAACAGCATTGCTGTCAC TT	
β-actin	CAGCCTTCCTTCTTGGG TATG	GGCATAGAGGTCTTTACGGAT G	

Supplementary Table 2: Primers used for transcript analysis in WT, *Ambn-IRESCre*$^{+/-}$, *Ambn-IRESCre*$^{+/+}$ and gene expression analysis (qPCR) in *Ambn-IRESCre*$^{+/-}$*/Smad4*$^{fl/fl}$ mice. All primers were purchased from Integrated DNA Technologies (IDT, USA). Primers were designed using IDT software where references are not listed. Primer set labeled Ambn# was only used for transcript analysis and not for qPCR.

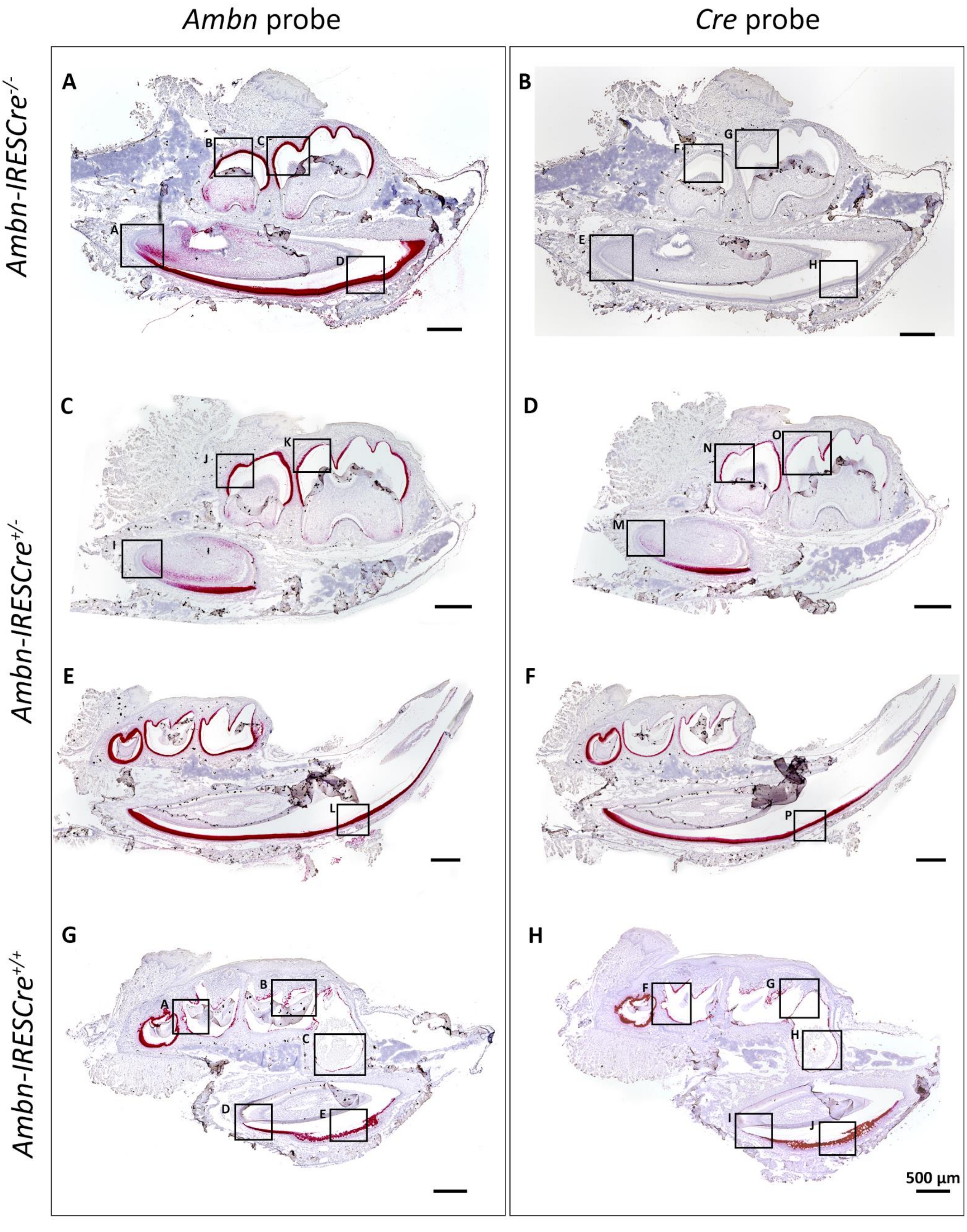

Ambn probe
Cre probe
Ambn-IRESCre-/-
Ambn-IRESCre+/-
Ambn-IRESCre+/+
A
B
C
D
E
F
G
H
500 µm

Supplementary Figure 2. Brightfield microscopic images of 14-days-old *Ambn-IRESCre*$^{-/-}$ (WT), *Ambn-IRESCre*$^{+/-}$, and *Ambn-IRESCre*$^{+/+}$ mandibles showing expression of *Ambn* (left column) and *Cre* (right column) RNA using *in situ* hybridization (RNAscope). In *Ambn-IRESCre*$^{-/-}$ or WT, Ambn expression was primarily in incisor and molar ameloblasts but also present in incisor pulp anterior to the cervical loop and near molar root apices (A). No non-specific background staining was observed when *Cre* probe was hybridized in WT samples (B). For *Ambn-IRESCre*$^{+/-}$, 2 sections were chosen to show RNA expression in the incisor cervical loops (C&D) and all 3 molars as well as the full length of the incisor (E&F). *Ambn* expression (C&E) remained unchanged in *Ambn-IRESCre*$^{+/-}$ as compared to WT. *Cre* expression closely mimicked *Ambn* expression pattern but was comparatively less abundant in the incisor pulp, molar root apices, and maturation stage ameloblasts (D&F). *Ambn-IRESCre*$^{+/+}$ incisor and molar ameloblasts were abnormal and interspersed by cysts positive for both *Ambn* and *Cre* (G&H). Black squares show regions selected for higher magnification images in Figures 4 and Supplementary Figure 8.

Ambn-IRESCre$^{+/-}$

Ambn probe — *Cre* probe

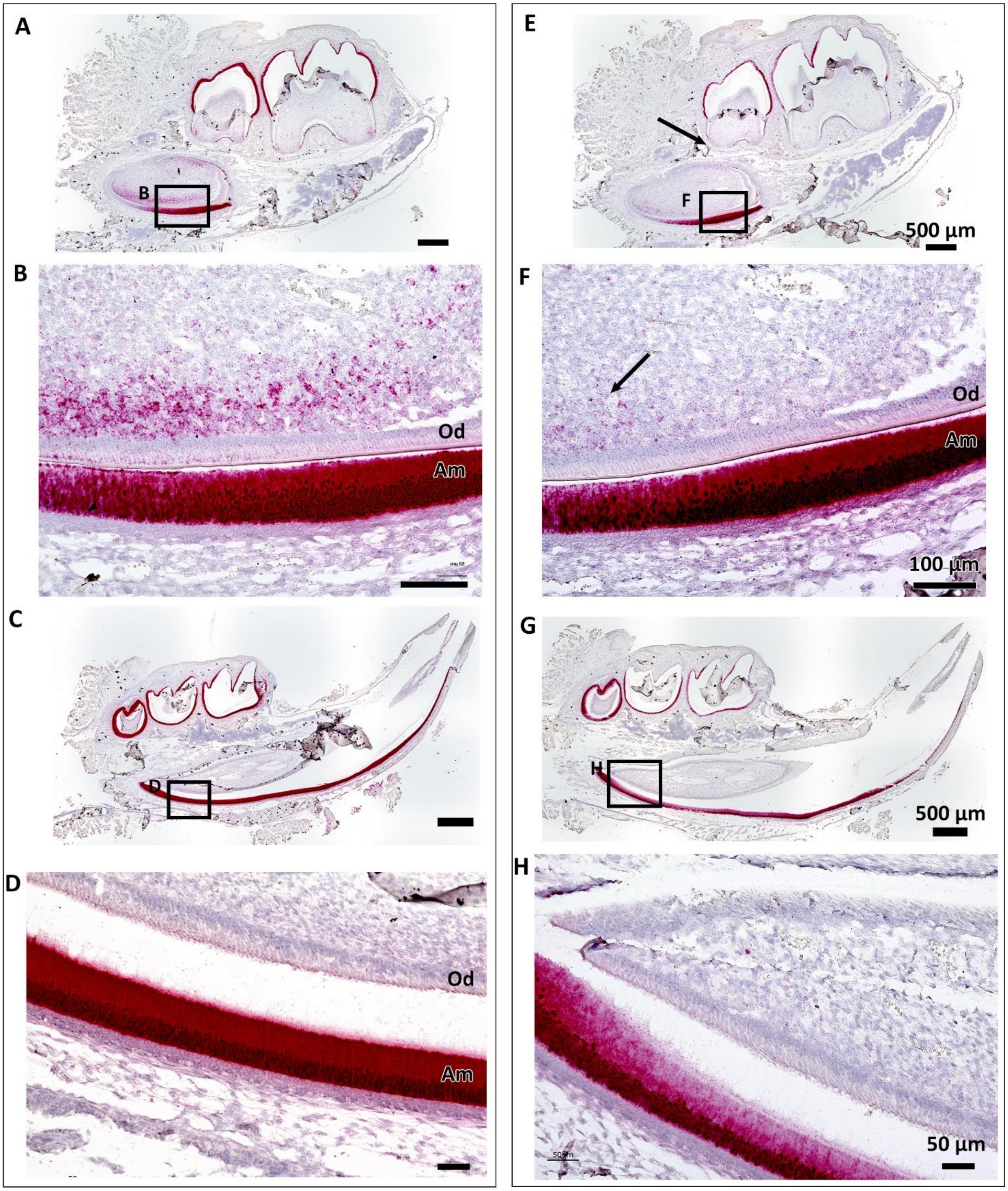

Supplementary Figure 3. Brightfield microscopic images showing *in situ* hybridization for *Ambn* and *Cre* RNA in PN14 *Ambn-IRESCre*$^{+/-}$ incisors*.* *Ambn* RNA (A-D) and *Cre* RNA (E-H). *Ambn-IRESCre*$^{+/-}$ odontoblasts show positive signal for *Ambn* in pre-odontoblasts and in the pulp cells superior to the odontoblasts (A&B). The early odontoblasts are also weakly positive for *Ambn* (B). Mature odontoblasts as well as the pulp cells lack similar *Ambn* signal (C&D). *Cre* RNA hybridization follows that of *Ambn* but the signal intensity is weaker in the pre-odontoblasts and pulp cells (E&F). Mature odontoblasts and pulp do not show any *Cre* labeling (G&H). Od: odontoblasts, Am: ameloblasts.

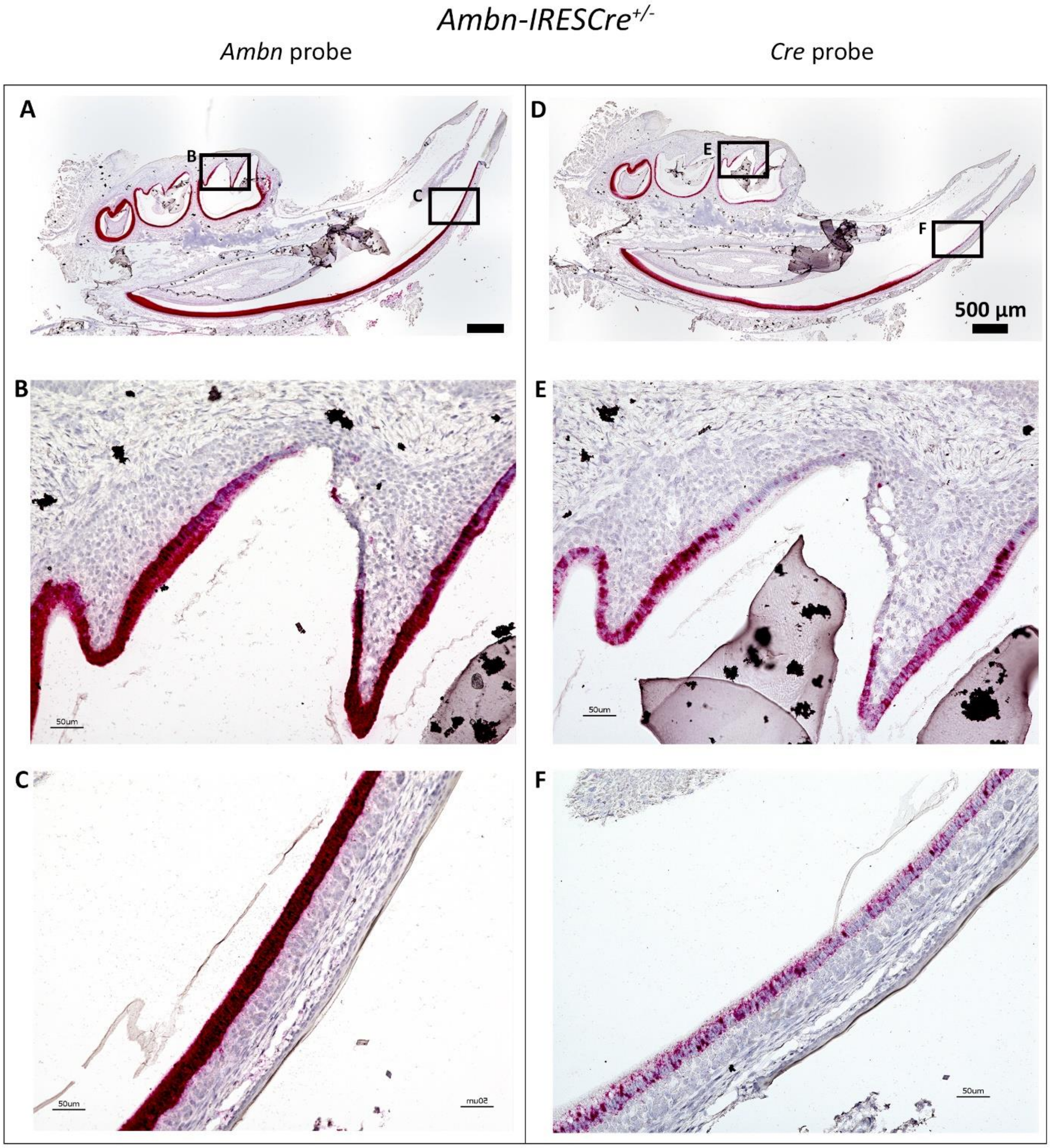

Supplementary Figure 4. Brightfield microscopic images showing *in situ* hybridization for *Ambn* and *Cre* RNA in PN14 *Ambn-IRESCre*$^{+/-}$ 1st molars. Robust *Ambn* signal is seen in late maturation stage 1st molar and incisor ameloblasts (A-C). However, *Cre* RNA is less abundant at this stage in the same region (D-F).

Ambn-IRESCre$^{+/+}$

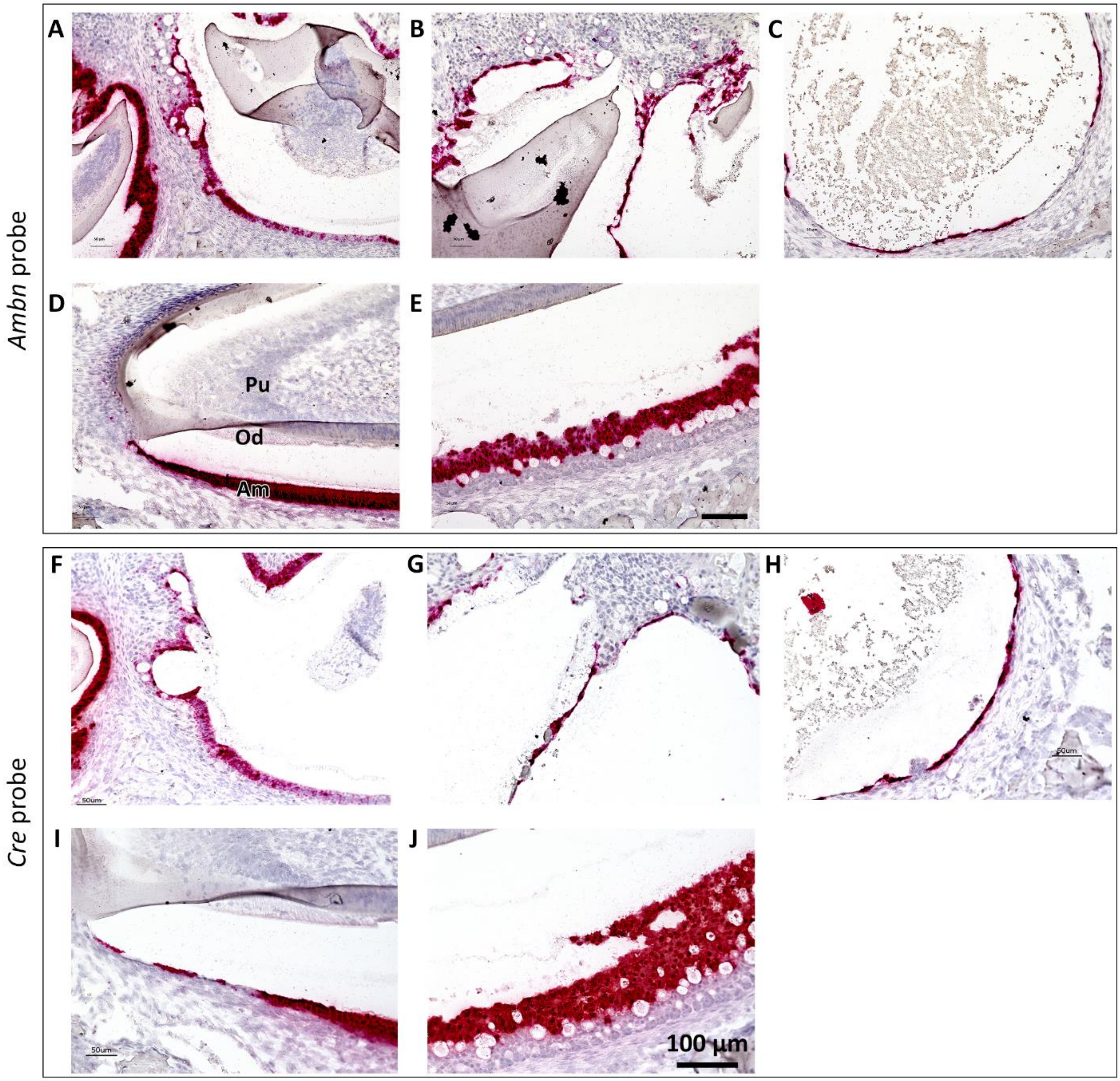

Supplementary Figure 5. Brightfield microscopic images showing *in situ* hybridization for *Ambn* (A-E) and *Cre* (F-J) in PN14 *Ambn-IRESCre*$^{+/+}$ incisor and molars. 2nd molar (A) and 1st molar (B) crowns show ameloblasts infiltrated with multiple small cysts positive for *Ambn*. A large cyst filled with amorphous contents is seen mesial to the first molar also lined with a thin layer of *Ambn* positive cells (C). Incisor ameloblast appear normal and have normal *Ambn* expression in early secretory stage (D) but eventually appear pathological with cysts (E) similar to the molars. *Cre* expression in molars (F&G), the 1st molar cyst (H) and incisors (I&J) closely follows *Ambn* expression in both intensity and localization. Low magnification images for these panels are in Supplementary Figure 5 G&H. Pu: pulp, Od: odontoblasts, Am: ameloblasts. Scale bar in J applies to all panels.

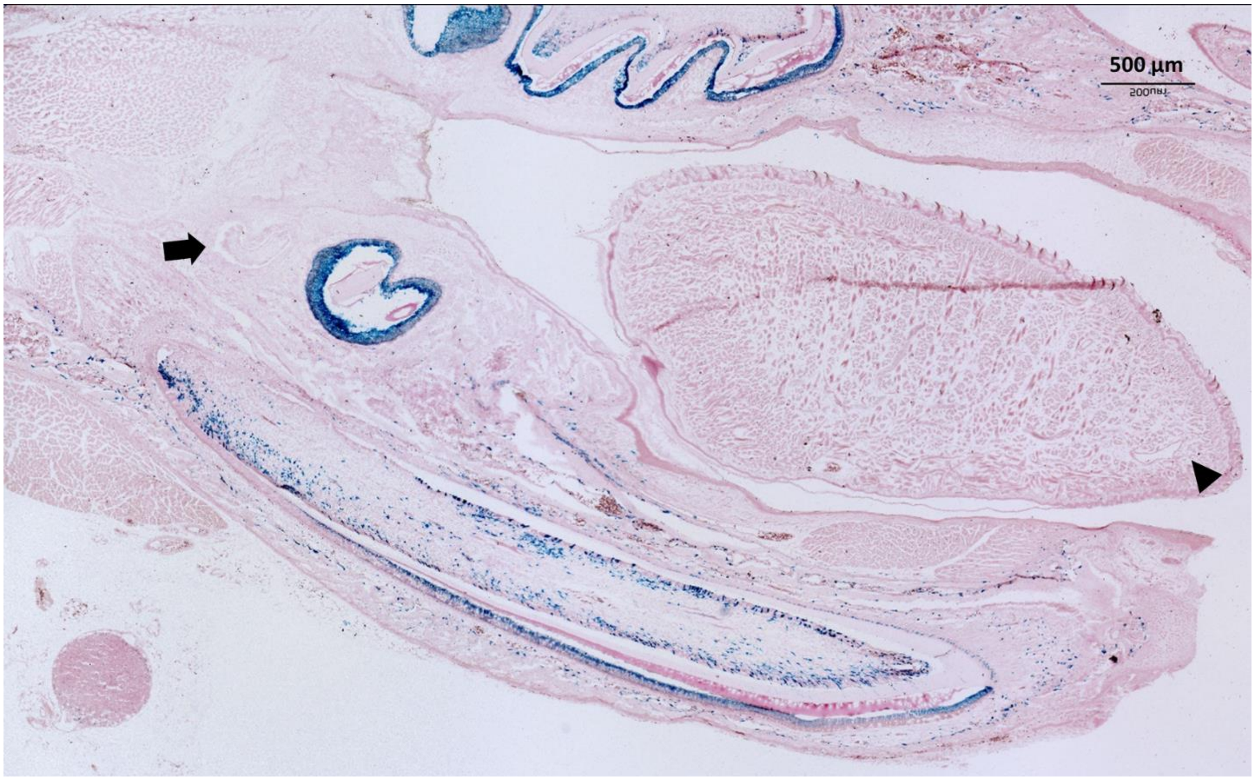

Supplementary Figure 6. Brightfield microscopic image of LacZ stained maxilla and mandible from *Ambn-IRESCre⁺/R26R⁺* mouse aged postnatal day 8 showing the expression pattern of Cre-recombinase (blue). Maxillary 1st and 2nd molars (top), mandibular 2nd molar (mandibular first molar is not in this section), and mandibular incisor ameloblasts were all LacZ positive; mandibular 3rd molar (indicated by black arrow) was LacZ negative. The tongue was also LacZ negative (black arrowhead). Scale bar in the top right corner.

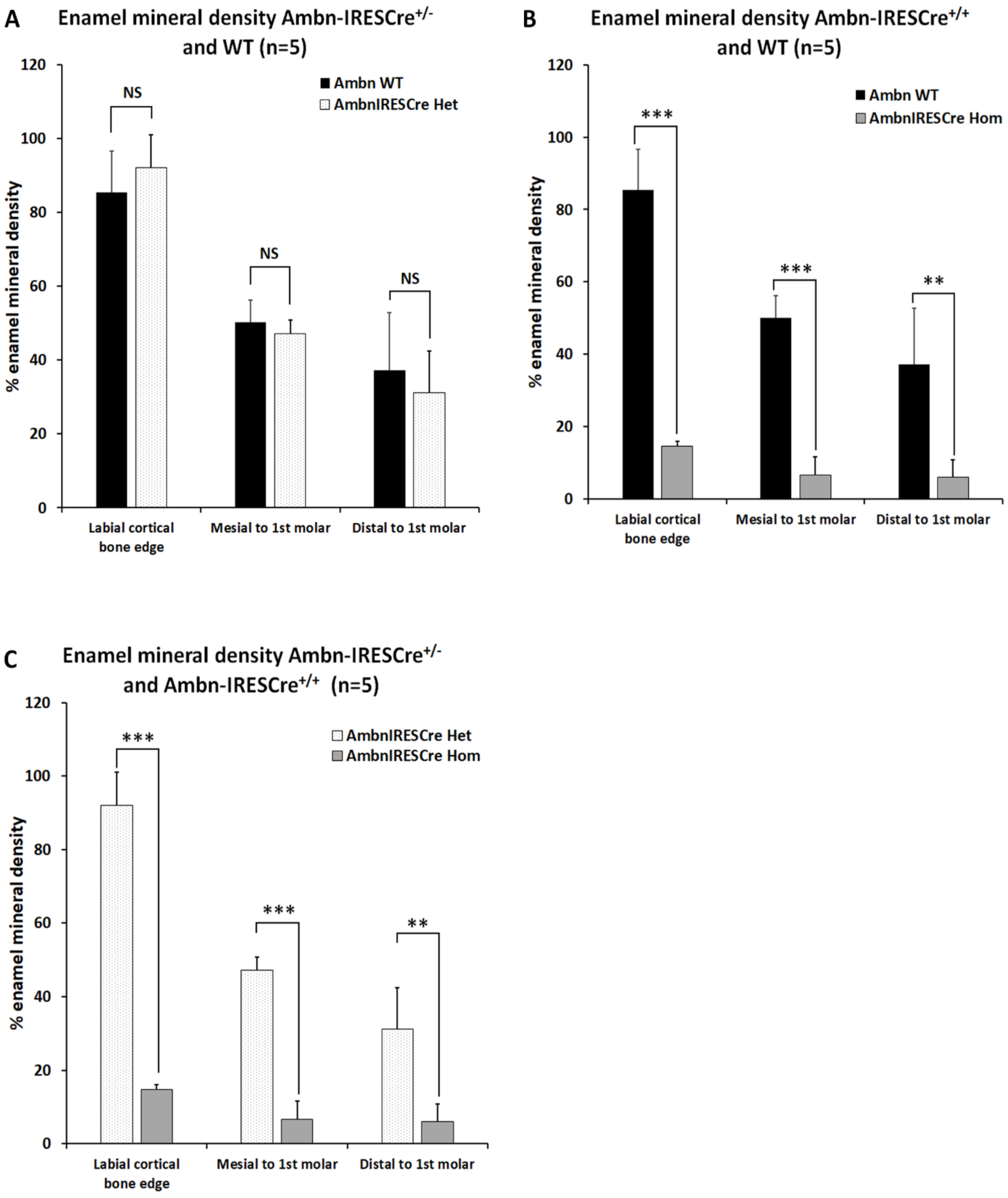
A
Enamel mineral density Ambn-IRESCre+/- and WT (n=5)
Ambn WT
AmbnIRESCre Het
% enamel mineral density
120
100
80
60
40
20
0
NS
NS
NS
Labial cortical bone edge
Mesial to 1st molar
Distal to 1st molar
B
Enamel mineral density Ambn-IRESCre+/+ and WT (n=5)
Ambn WT
AmbnIRESCre Hom
% enamel mineral density
120
100
80
60
40
20
0
***
***
**
Labial cortical bone edge
Mesial to 1st molar
Distal to 1st molar
C
Enamel mineral density Ambn-IRESCre+/- and Ambn-IRESCre+/+ (n=5)
AmbnIRESCre Het
AmbnIRESCre Hom
% enamel mineral density
120
100
80
60
40
20
0
***
***
**
Labial cortical bone edge
Mesial to 1st molar
Distal to 1st molar

Supplementary Figure 7. Statistical analysis of relative incisor enamel mineral densities in 8-weeks-old WT, *Ambn-IRESCre*$^{+/-}$ and *Ambn-IRESCre*$^{+/+}$ mice. Comparison between WT and *Ambn-IRESCre*$^{+/-}$ (A); WT and *Ambn-IRESCre*$^{+/+}$ (B); and *Ambn-IRESCre*$^{+/-}$ and *Ambn-IRESCre*$^{+/+}$ (C) showing mean relative incisor enamel mineral density values with standard deviation. No significant differences were observed between WT and *Ambn-IRESCre*$^{+/-}$ relative enamel mineral density (A). But *Ambn-IRESCre*$^{+/+}$ incisor enamel was severely hypomineralized as compared to both WT (B) and *Ambn-IRESCre*$^{+/-}$ (C). Statistical analysis performed by unpaired two-sample t-test assuming unequal variances; n=5 mice; * $p \le 0.05$, ** $p \le 0.01$, *** $p \le 0.001$.

Ambn-IRESCre$^{+/+}$

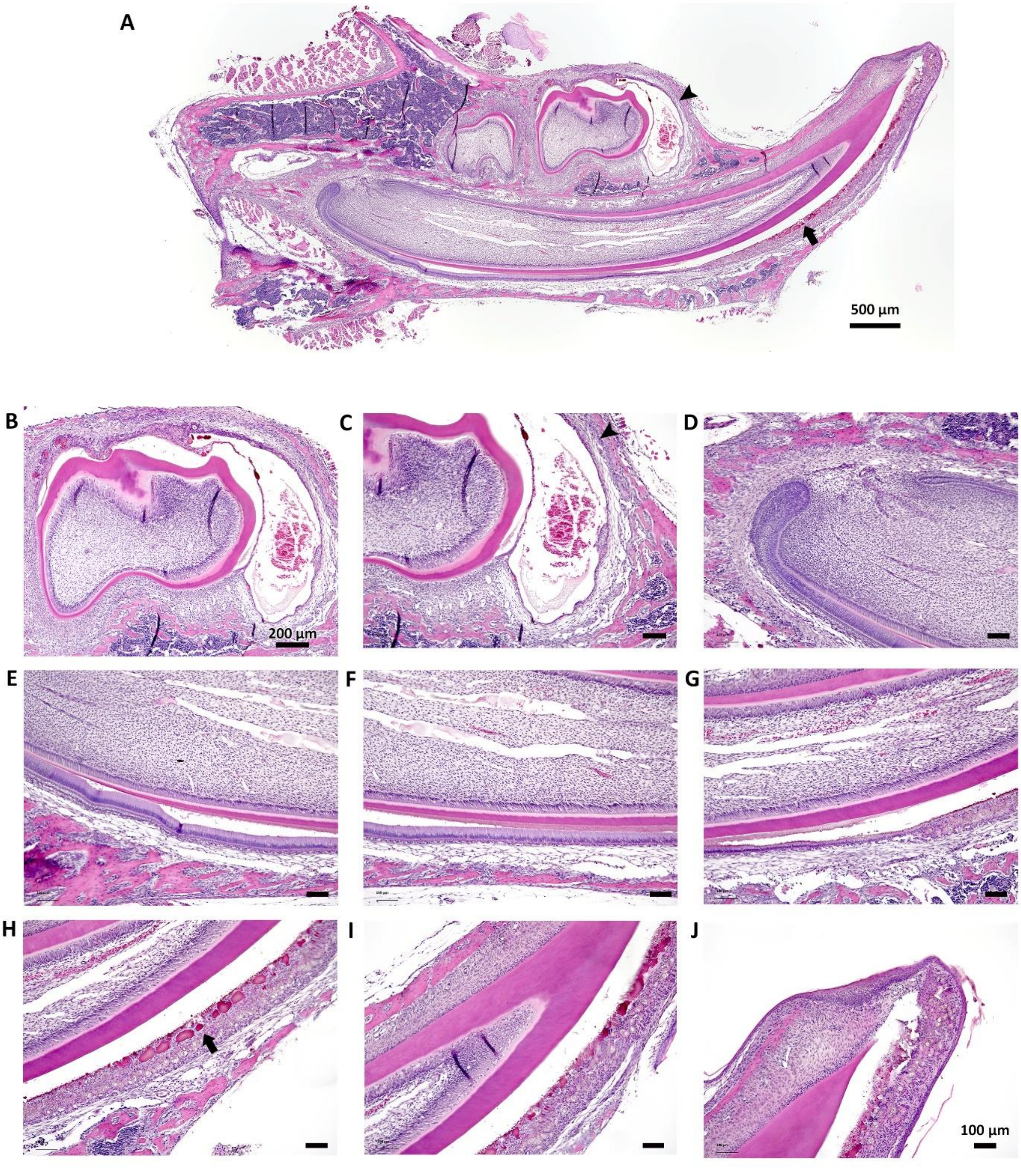

Supplementary Figure 8. Brightfield microscopic image showing 14-days-old hematoxylin and eosin (H&E) stained *Ambn-IRESCre*$^{+/+}$ mandible (A). Cyst mesial to the 1st molar marked by black arrowhead and dysmorphic incisor ameloblasts marked by black arrow (A). Higher magnification image of the 1st molar and associated cyst (B, C) with cyst lining marked with an arrowhead (C). Higher magnification images of the incisor ameloblast starting from the cervical loop (D) to the incisor tip (J). The cervical loop and secretory stage incisor ameloblasts (D-F) do not show gross morphological defects at this magnification until transition stage (G). In transition and maturation stages ameloblasts appear dysmorphic (G-J) and amorphous material accumulates within the ameloblast layer (arrow, H). Scale bar for C-J is 100 µm.

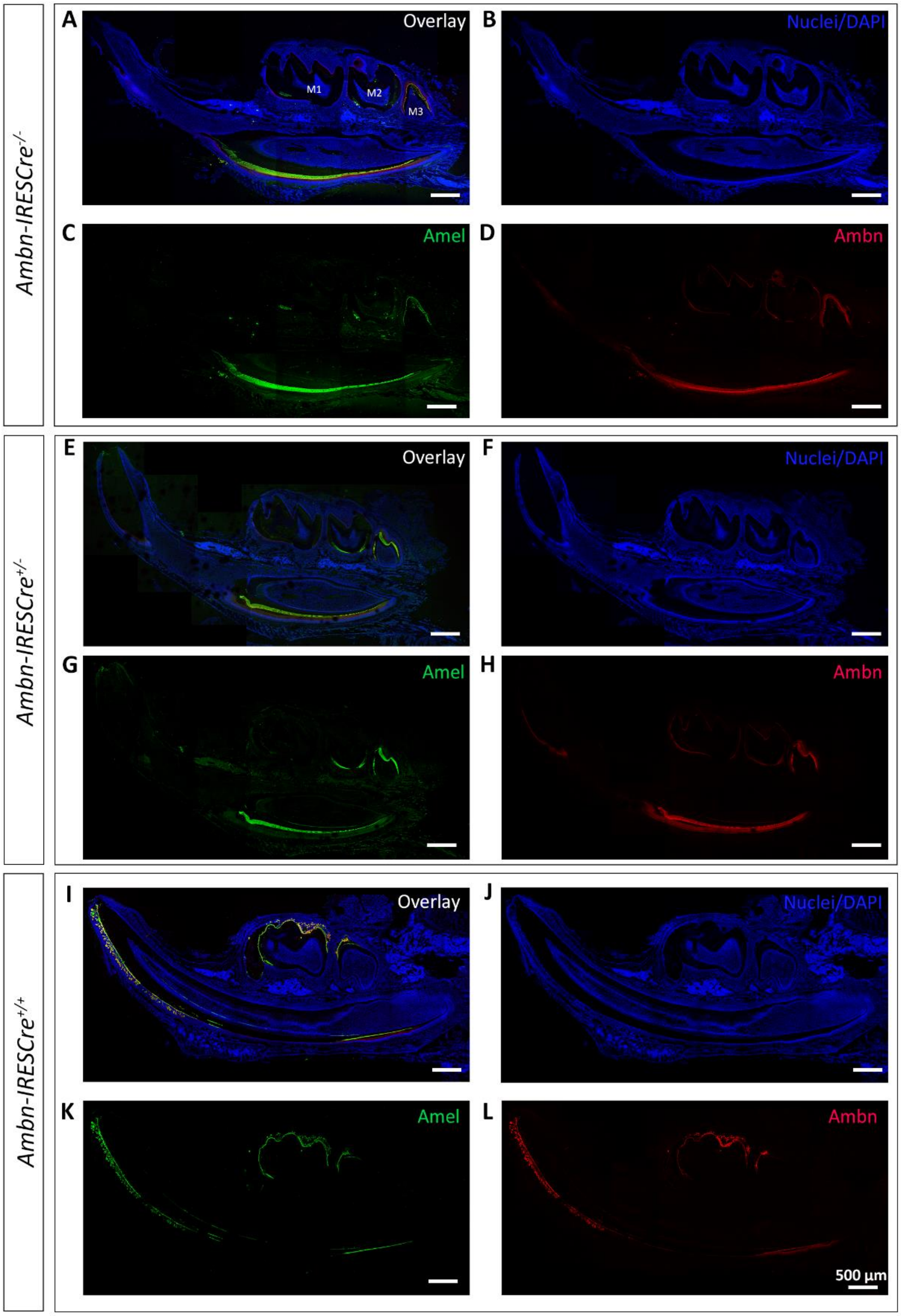

Ambn-IRESCre-/-
A
Overlay
M1
M2
M3
B
Nuclei/DAPI
C
Amel
D
Ambn
Ambn-IRESCre+/-
E
Overlay
F
Nuclei/DAPI
G
Amel
H
Ambn
Ambn-IRESCre+/+
I
Overlay
J
Nuclei/DAPI
K
Amel
L
Ambn
500 µm

Supplementary Figure 9. Confocal microscopic images of PN14 *Ambn-IRESCre*$^{-/-}$ (WT, A-D), *Ambn-IRESCre*$^{+/-}$ (E-H) and *Ambn-IRESCre*$^{+/+}$ (I-L) mandibles co-labeled with anti-amelogenin (green) and anti-ameloblastin (red) antibodies. Amelogenin (C, G and K) and ameloblastin (D, H and L) were expressed by ameloblasts in incisors and molars of all three genotypes.

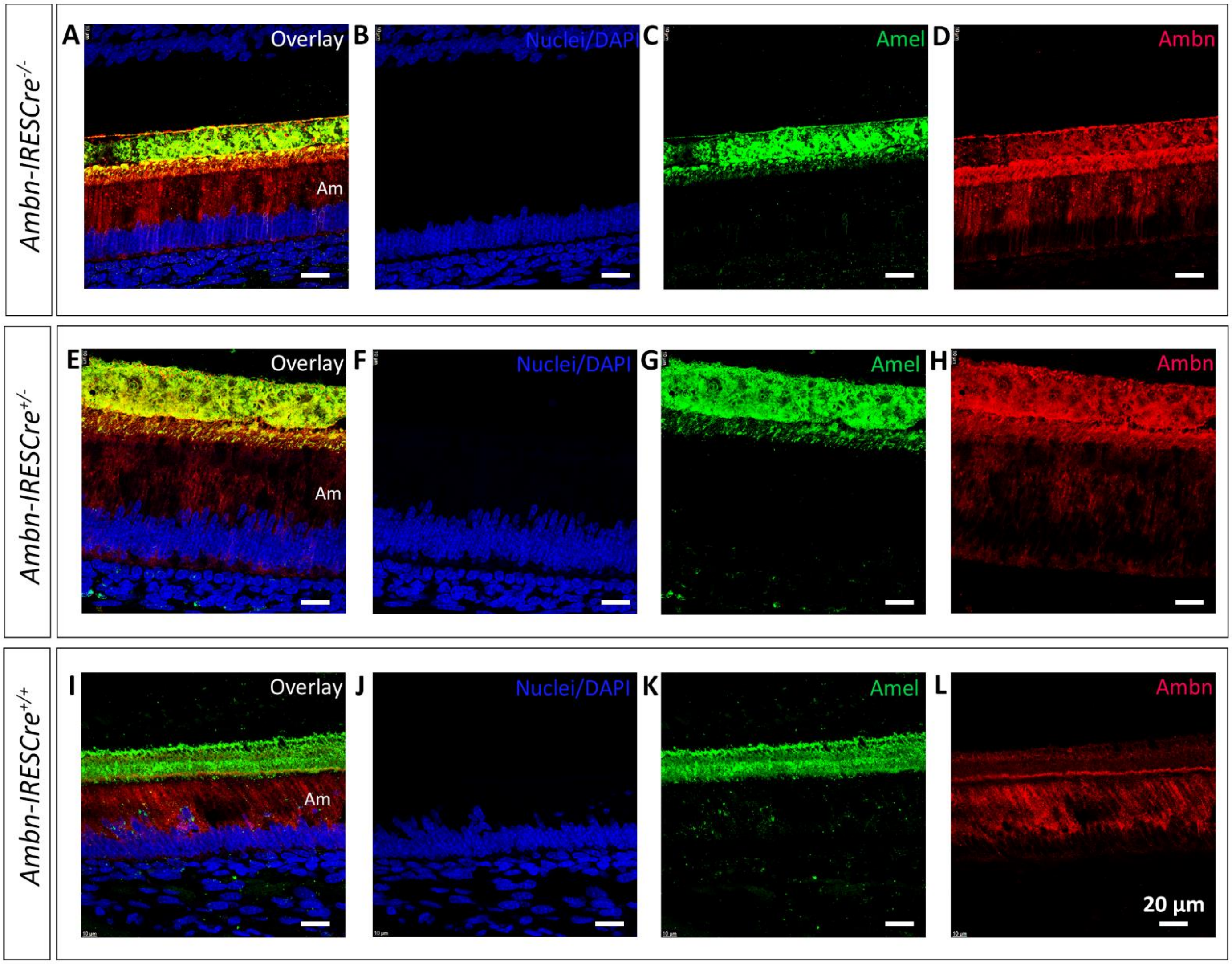

Supplementary Figure 10. Higher magnification confocal microscopic images of PN14 *Ambn-IRESCre*$^{-/-}$ (WT, A-D), *Ambn-IRESCre*$^{+/-}$ (E-H) and *Ambn-IRESCre*$^{+/+}$ (I-L) ameloblasts in secretory stage co-labeled with anti-amelogenin (green) and anti-ameloblastin (red) antibodies. Amelogenin (C, G and K) and ameloblastin (D, H and L) were expressed by all three genotypes. However, as compared to WT (D) and *Ambn-IRESCre*$^{+/-}$ (H), less ameloblastin appeared in the *Ambn-IRESCre*$^{+/+}$ enamel extracellular matrix (L) and more within the ameloblasts. Am: ameloblasts.

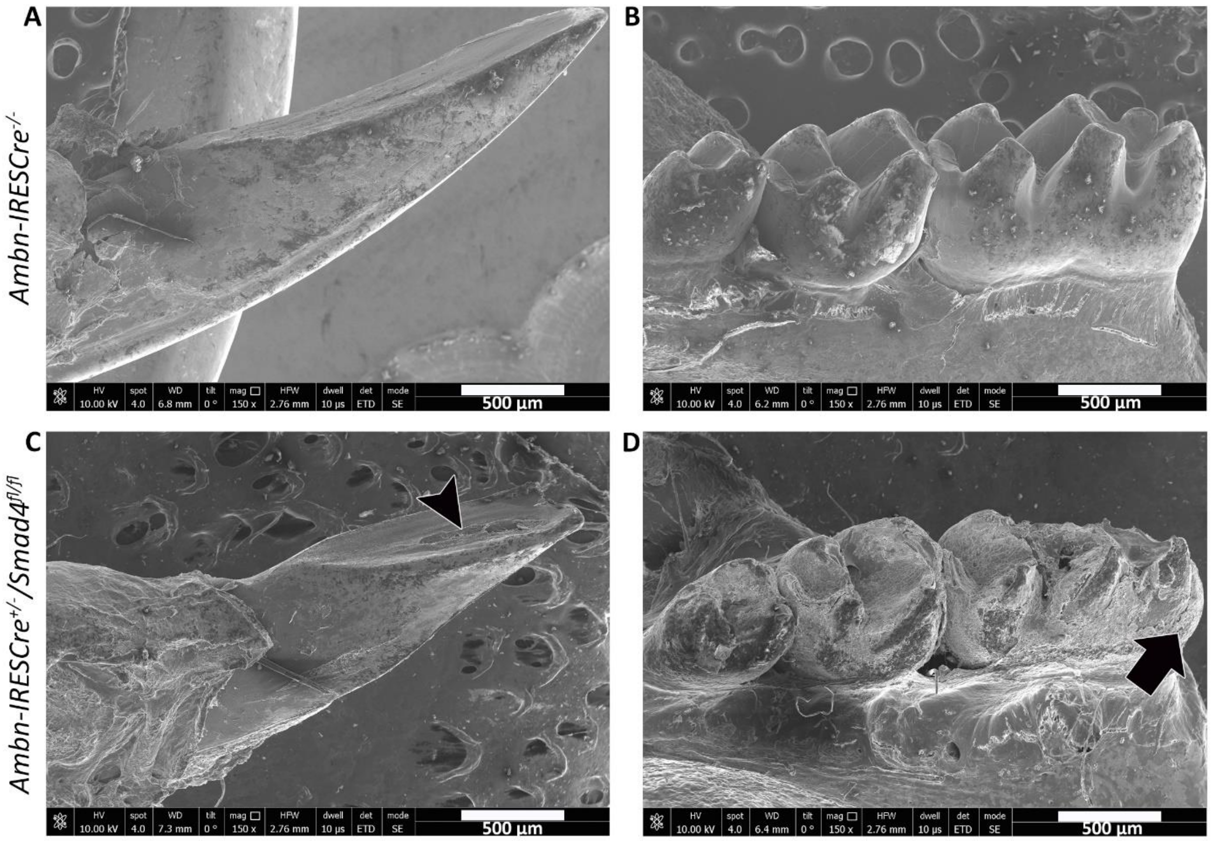

Supplementary Figure 11. Scanning electron microscopic images of 8-weeks-old WT (A&B) and *Ambn-IRESCre*$^{+/-}$*/Smad4*$^{fl/fl}$ (C&D) incisors and molars. Wild type incisor and molars (A&B) showing normal uniform layer of enamel covering the labial surface of incisor (A) and crowns of all 3 molars (B). Certain *Ambn-IRESCre*$^{+/-}$*/Smad4*$^{fl/fl}$ incisors showed pulp exposure (C, black arrowhead). *Ambn-IRESCre*$^{+/-}$*/Smad4*$^{fl/f}$ molar occlusal enamel was worn and fractured away from underlying dentin and intact enamel persisted only around the cervical third of the crown (D, black arrow).

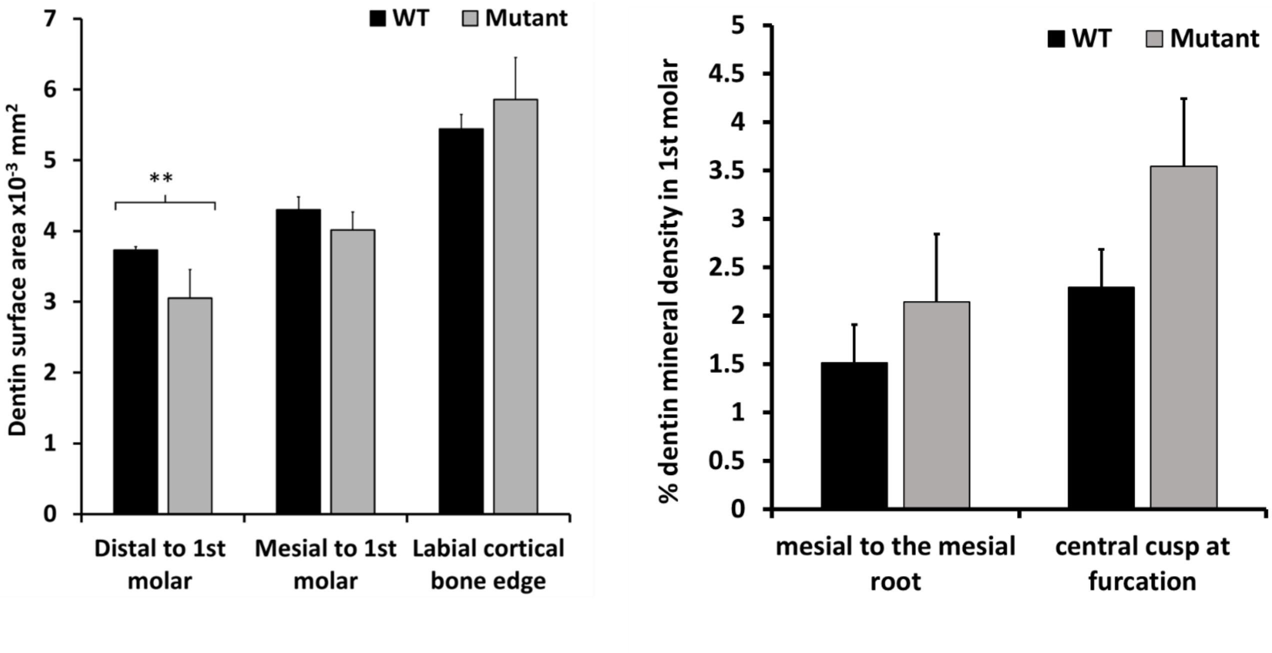

Supplementary Figure 12. At distal to the 1st molar region, *Ambn-IRESCre*$^{+/-}$*/Smad4*$^{fl/fl}$ dentin surface area was significantly lower than that of WT (A), which appeared as enlarged pulp chamber (Figure 7 N). There were no significant differences compared to WT in the dentin surface area of 8-week-old *Ambn-IRESCre*$^{+/-}$*/Smad4*$^{fl/fl}$ incisors measured at the mesial to the 1st molar region and labial cortical bone edge (A); n = 6 mice. The 1st molar dentin mineral density of 8-week-old *Ambn-IRESCre*$^{+/-}$*/Smad4*$^{fl/fl}$ mutants was higher as compared to WT but the change was not statistically significant (B) n = 3 mice, 6 data points. Statistical analysis performed by unpaired 2-sample t-tests assuming unequal variances. ** $p \leq 0.01$

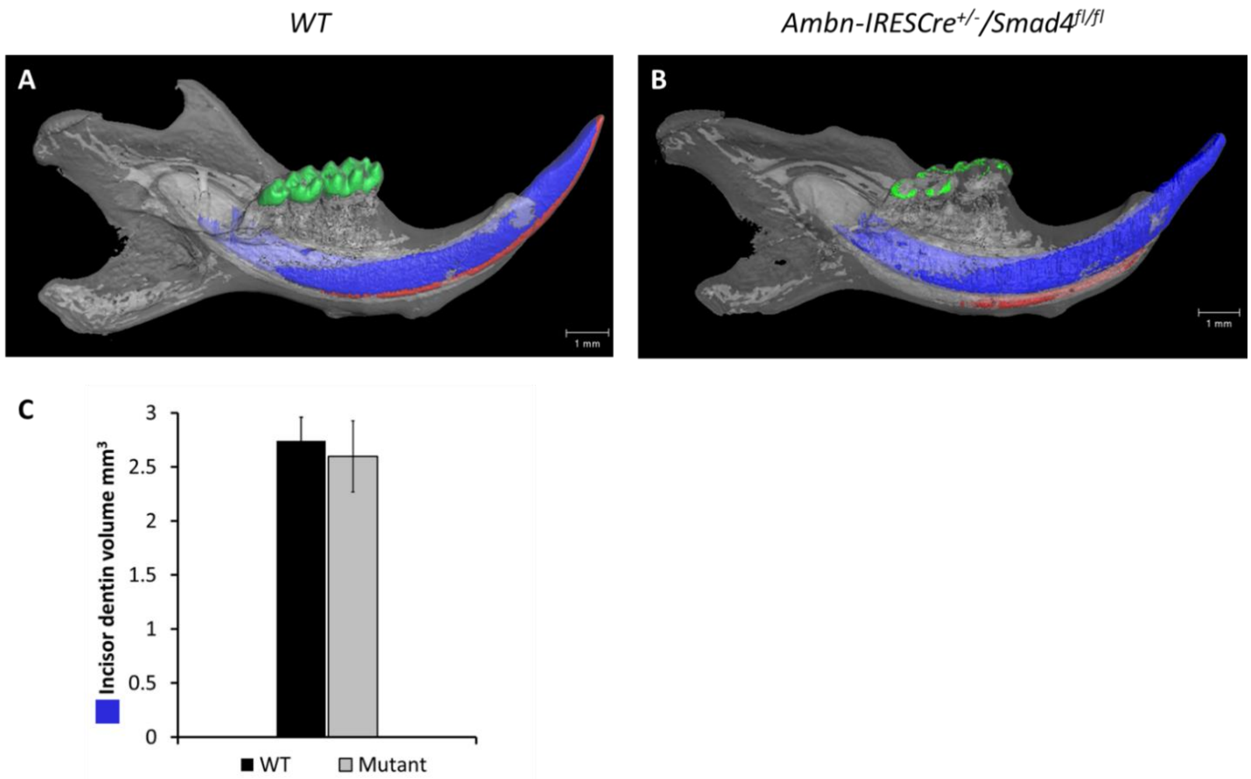

Supplementary Figure 13. Three-dimensional µCT reconstruction of 8-week-old WT (A) and *Ambn-IRESCre*$^{+/-}$*/Smad4*$^{fl/fl}$ (B) mandibles with incisor dentin volume pseudo-colored in blue, incisor enamel in red, and molar enamel in green. Graph representing mean incisor dentin volume with standard deviation (C) shows no significant difference ($p > 0.05$) between the WT and mutant. Statistical analysis performed by unpaired 2-sample t-test assuming unequal variances. N= 6 mice.

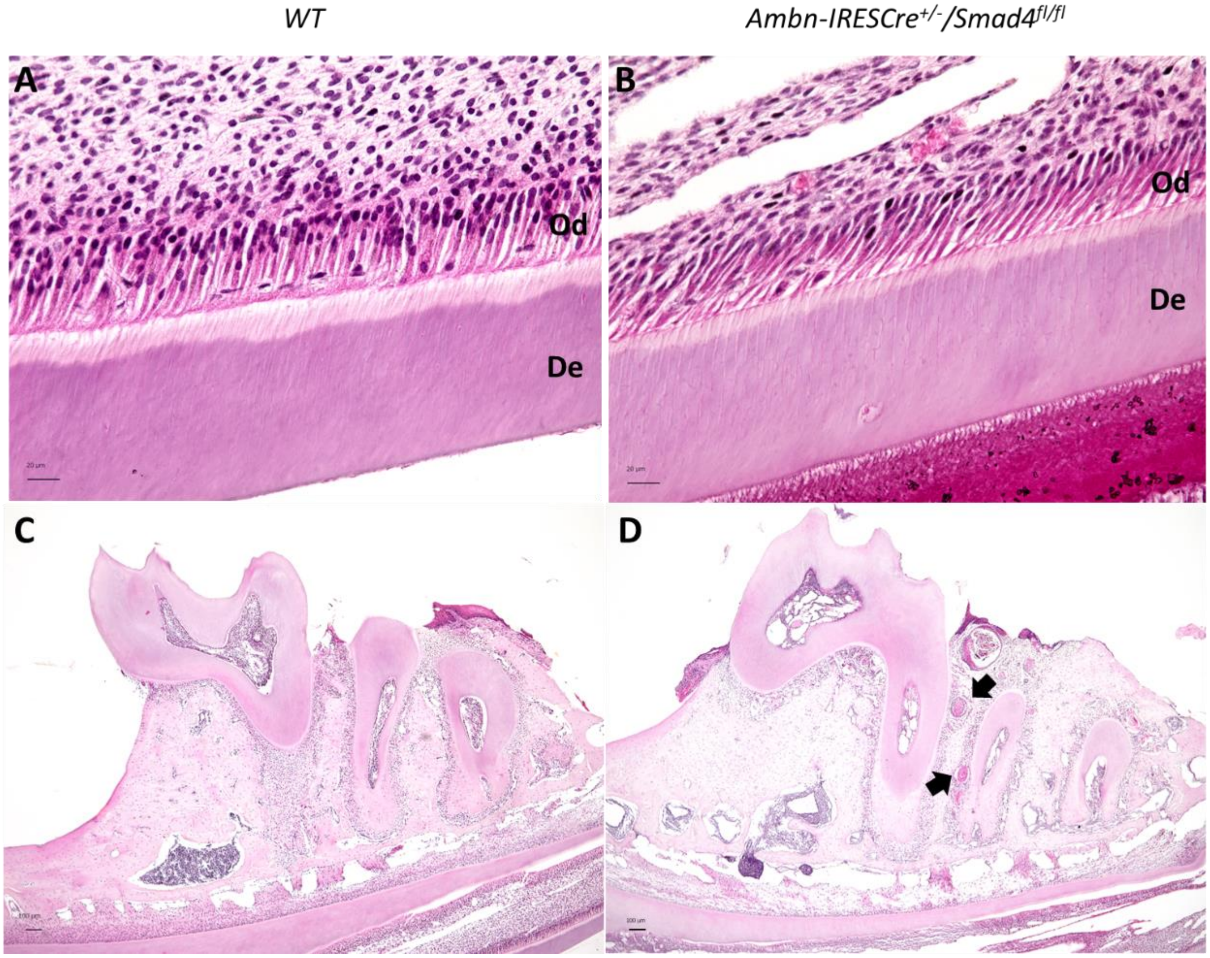

Supplementary Figure 14. Brightfield microscopic images of 8-weeks-old hematoxylin and eosin (H&R) stained WT and *Ambn-IRESCre*$^{+/-}$*/Smad4*$^{fl/fl}$ odontoblasts, dentin and molar roots. No differences were observed between WT (A) and *Ambn-IRESCre*$^{+/-}$*/Smad4*$^{fl/fl}$ (B) odontoblasts and dentin. *Ambn-IRESCre*$^{+/-}$*/Smad4*$^{fl/fl}$ molar roots (D) also appeared normal except the presence of enamel pearls (D, black arrows) between first and second molar roots. Od: odontoblasts, De: dentin.

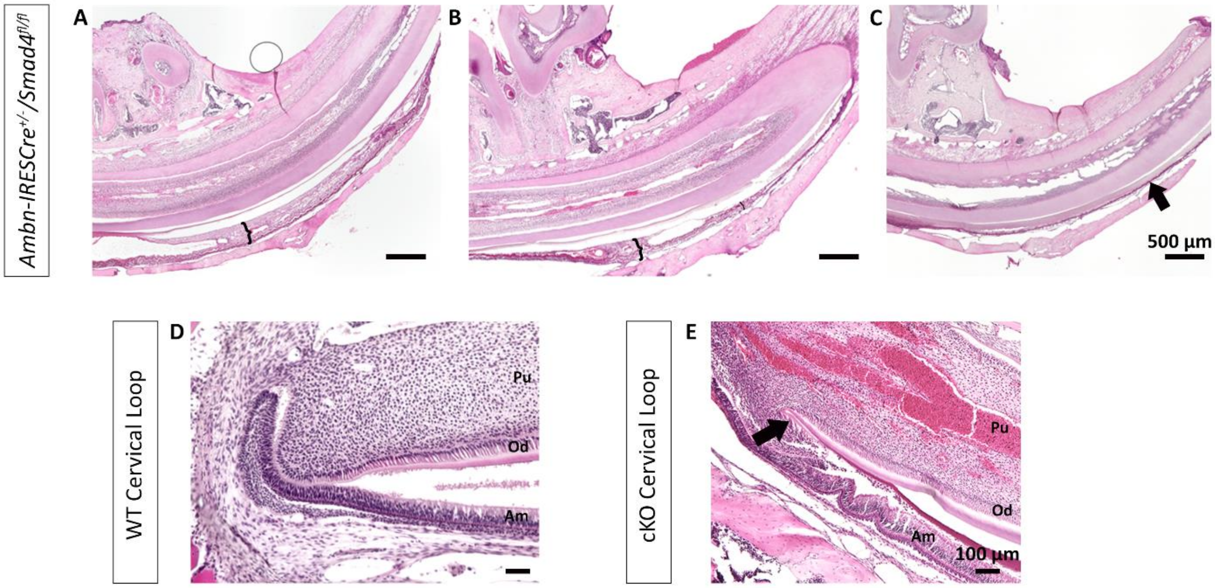

Supplementary Figure 15. Brightfield microscopic images of 8-week-old H&E stained mandibles from three different *Ambn-IRESCre*$^{+/-}$*/Smad4*$^{fl/fl}$ mutant mice showing differences in the thickness of the layer of spindle-like cells infiltrating the enamel organ. This layer is > 100 µm thick and marked by braces in A&B but is very thin in C and marked by a black arrow. Normal incisor cervical loop in WT (D). *Ambn-IRESCre*$^{+/-}$*/Smad4*$^{fl/fl}$ mutant cervical loop showing pathological ameloblasts (E). Pu: pulp, Od: odontoblasts, Am: ameloblasts.

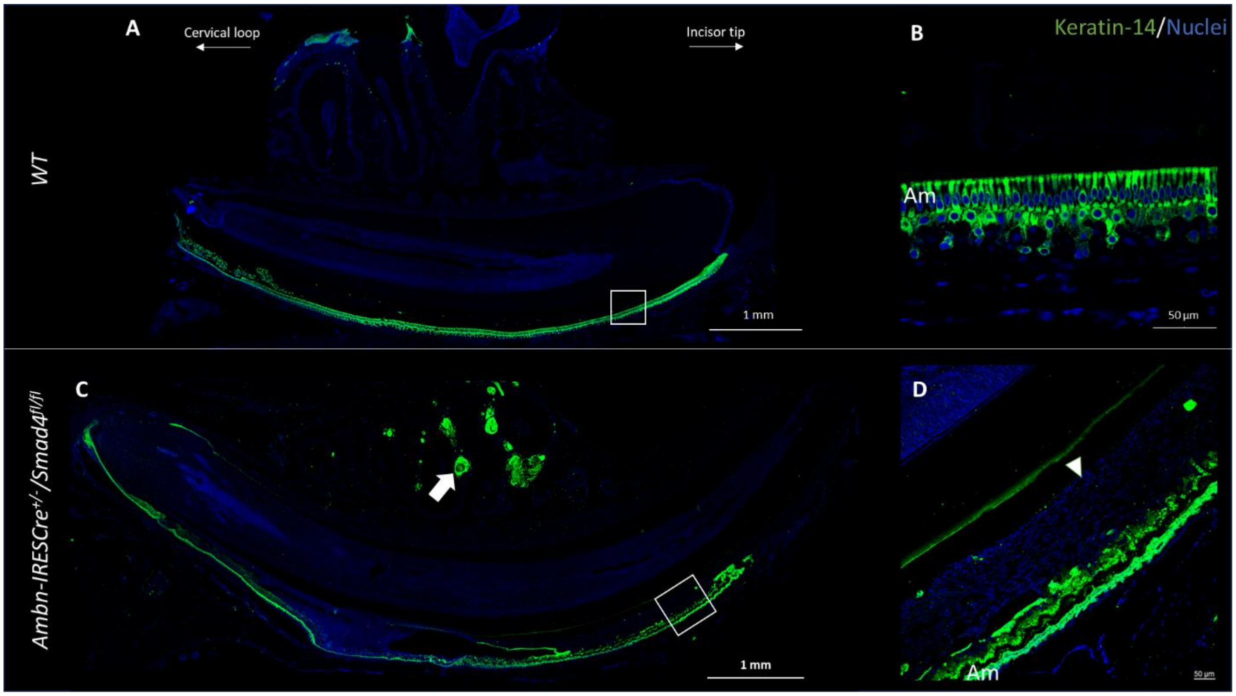

Supplementary Figure 16. Confocal images showing immunofluorescence for cytokeratin-14 (green) specifically labeling ameloblasts and papillary layer in both wildtype (A and B) and *Ambn-IRESCre*$^{+/-}$*/Smad4*$^{fl/fl}$ incisors (C and D). Higher magnification image of WT ameloblasts show apical ends of ameloblasts and stratum intermedium cells positive for Keratin-14 (B). Enamel pearls near mutant molar roots are also positive for Keratin-14 (white arrow in C). Higher magnification image mutant ameloblasts show the ameloblast layer is irregular and the layer of spindle shaped cells infiltrating the mutant enamel organ does not express Keratin-14 (white arrowhead in D). Am: ameloblasts.

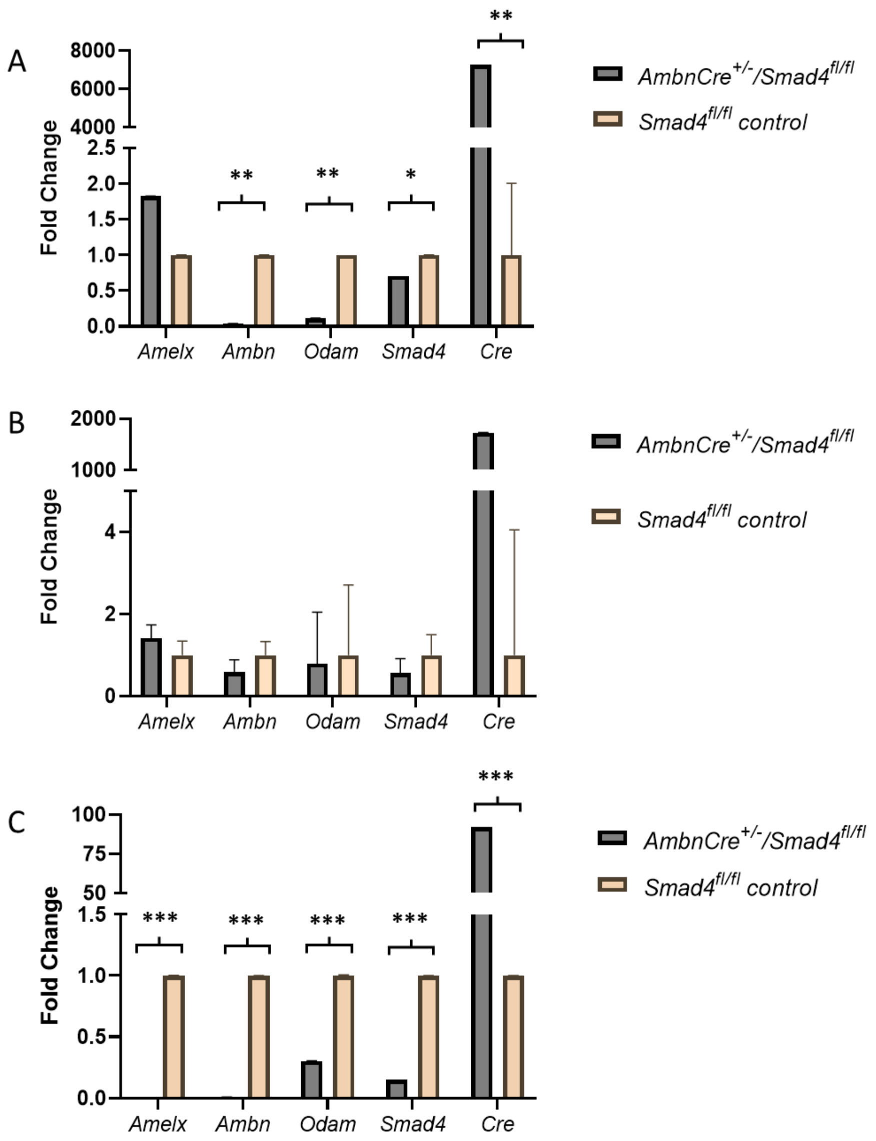

Supplementary Figure 17. Selected gene profiling from PN14 mandibular first molar enamel organs of three *Ambn-IRESCre*$^{+/-}$/*Smad4*$^{fl/fl}$ mice (biological replicates). Real time quantitative PCR data depicted as fold change ($2^{-\Delta\Delta Ct}$) showing downregulation of *Ambn*, *Odam*, and *Smad4* in all three samples. *Cre* is only expressed in the mutants and the Ct values for WT *Cre* are either not detected or between 37-40 cycles. Statistical analysis performed by unpaired two-sample t-test assuming unequal variances. N = 4 technical replicates * p ≤ 0.05, ** p ≤ 0.01, *** p ≤ 0.001.

References

Lacruz RS, Hacia JG, Bromage TG, Boyde A, Lei Y, Xu Y, Miller JD, Paine ML, Snead ML. 2012. The circadian clock modulates enamel development. J Biol Rhythms. 27(3):237-45.

Sakai M, Troutman TD, Seidman JS, Ouyang Z, Spann NJ, Abe Y, Ego KM, Bruni CM, Deng Z, Schlachetzki JC, Nott A. et al. 2019. Liver-derived signals sequentially reprogram myeloid enhancers to initiate and maintain Kupffer cell identity. Immunity. d51(4):655-70.
